# An evolved pyrrolysyl-tRNA synthetase with polysubstrate specificity expands the toolbox for engineering enzymes with incorporation of noncanonical amino acids

**DOI:** 10.1186/s40643-023-00712-w

**Published:** 2023-12-11

**Authors:** Ke Liu, Ling Jiang, Shuang Ma, Zhongdi Song, Lun Wang, Qunfeng Zhang, Renhao Xu, Lirong Yang, Jianping Wu, Haoran Yu

**Affiliations:** 1 Institute of Bioengineering, College of Chemical and Biological Engineering, Zhejiang University, Hangzhou, 310027 Zhejiang China; 2 ZJU-Hangzhou Global Scientific and Technological Innovation Centre, Hangzhou, 311200 Zhejiang China; 3 Key Laboratory of Pollution Exposure and Health Intervention of Zhejiang Province, Interdisciplinary Research Academy, Zhejiang Shuren University, Hangzhou, 310015 Zhejiang China; 4 Hangzhou 14th Middle School, Hangzhou, 310006 Zhejiang China

**Keywords:** Pyrrolysyl-tRNA synthetase, Genetic code expansion, Noncanonical amino acid, Protein main-chain modification, PedH

## Abstract

Aminoacyl-tRNA synthetase (aaRS) is a core component for genetic code expansion (GCE), a powerful technique that enables the incorporation of noncanonical amino acids (ncAAs) into a protein. The aaRS with polyspecificity can be exploited in incorporating additional ncAAs into a protein without the evolution of new, orthogonal aaRS/tRNA pair, which hence provides a useful tool for probing the enzyme mechanism or expanding protein function. A variant (N346A/C348A) of pyrrolysyl-tRNA synthetase from *Methanosarcina mazei* (*Mm*PylRS) exhibited a wide substrate scope of accepting over 40 phenylalanine derivatives. However, for most of the substrates, the incorporation efficiency was low. Here, a *Mb*PylRS (N311A/C313A) variant was constructed that showed higher ncAA incorporation efficiency than its homologous *Mm*PylRS (N346A/C348A). Next, N-terminal of *Mb*PylRS (N311A/C313A) was engineered by a greedy combination of single variants identified previously, resulting in an IPE (N311A/C313A/V31I/T56P/A100E) variant with significantly improved activity against various ncAAs. Activity of IPE was then tested toward 43 novel ncAAs, and 16 of them were identified to be accepted by the variant. The variant hence could incorporate nearly 60 ncAAs in total into proteins. With the utility of this variant, eight various ncAAs were then incorporated into a lanthanide-dependent alcohol dehydrogenase PedH. Incorporation of phenyllactic acid improved the catalytic efficiency of PedH toward methanol by 1.8-fold, indicating the role of modifying protein main chain in enzyme engineering. Incorporation of *O*-tert-Butyl-L-tyrosine modified the enantioselectivity of PedH by influencing the interactions between substrate and protein. Enzymatic characterization and molecular dynamics simulations revealed the mechanism of ncAAs affecting PedH catalysis. This study provides a PylRS variant with high activity and substrate promiscuity, which increases the utility of GCE in enzyme mechanism illustration and engineering.

**Graphical Abstract:**

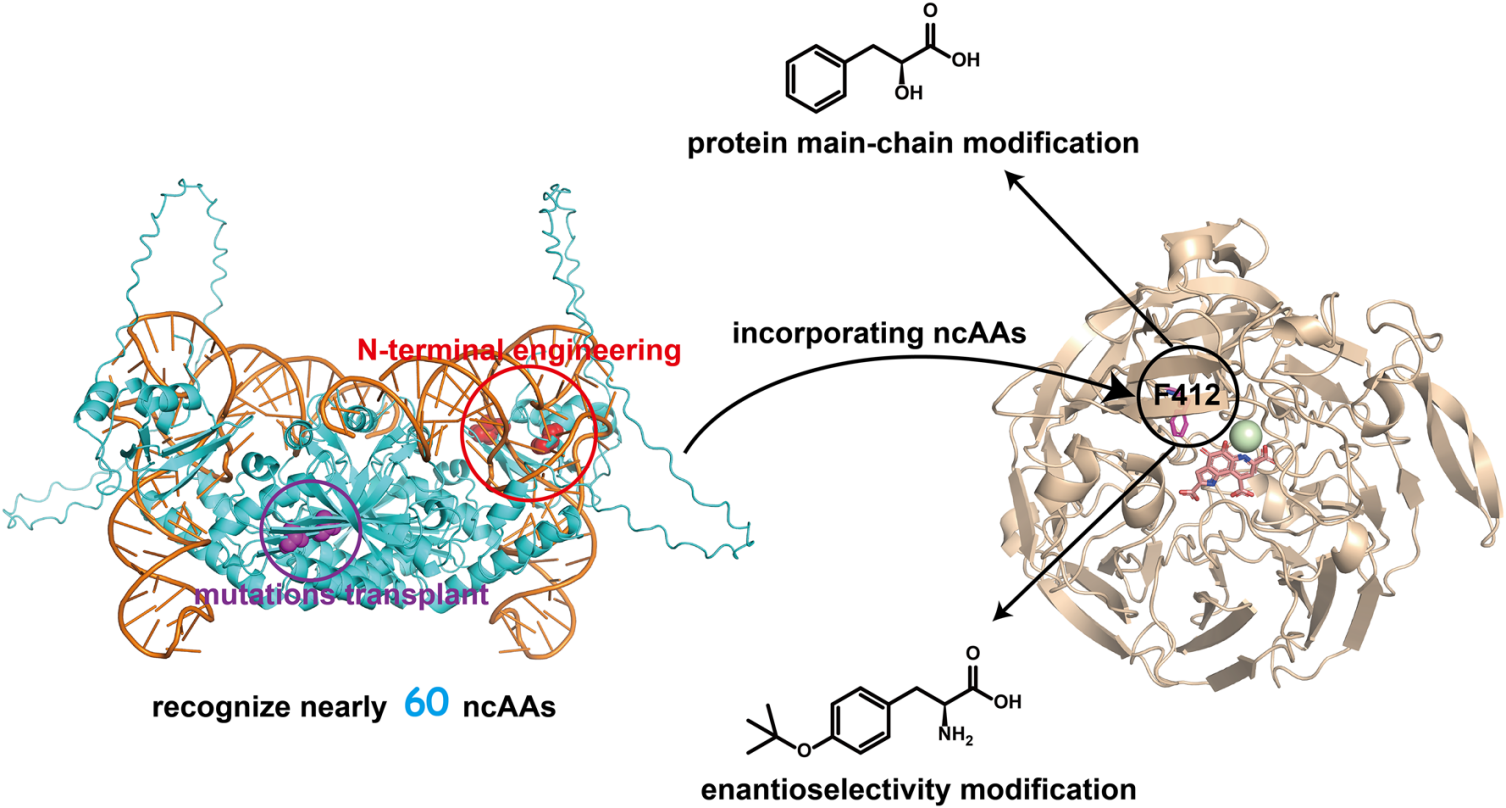

**Supplementary Information:**

The online version contains supplementary material available at 10.1186/s40643-023-00712-w.

## Introduction

Genetic code expansion is a powerful tool that enables the incorporation of noncanonical amino acids (ncAAs) into a protein at specific positions (Shandell et al. 2021). Generally, it relies on the suppression of an amber stop codon (UAG) by a suppressor tRNA coupled with an evolved aminoacyl-tRNA synthetase (aaRS). The *Methanocaldococcus jannaschii* tyrosyl-tRNA synthetase (*Mj*TyrRS)/tRNA_CUA_ pair and the pyrolysyl-tRNA synthetase (PylRS)/tRNA_CUA_ pair from both *Methanosarcina barkeri* and *Methanosarcina mazei* are most widely used for genetic code expansion in bacteria and a variety of eukaryotes including mammalian cells and multicellular organism (Brown et al. 2018). The directed evolution of the aaRS has generated numerous variants that enabled incorporation of over 200 ncAAs with diverse chemical and physical properties into proteins (Wan et al. 2014). The incorporation of a new ncAA typically requires selection of large libraries of aaRS with randomized active site mutations. A powerful method has been developed for the directed evolution of aaRS involving both positive and negative selections based on the ability to suppress a nonsense mutation in the presence of the desired ncAA (Bryson et al. 2017). However, this process requires multiple rounds of selection for each new substrate, which is time-consuming and laborious.

Since no selection pressure was applied toward other ncAAs during directed evolution, evolved aaRSs may also cross-react with other ncAAs, while maintaining their orthogonality to endogenous amino acids. This polyspecificity can hence be exploited in incorporating additional ncAAs without the evolution of new, orthogonal aaRS/tRNA pairs. For example, the engineered aaRS *p*CNFRS specific for *p*-cyano-L-phenylalanine (*p*CNF) derived from *Methanocaldococcus jannaschii* tyrosyl-tRNA synthetase can recognize at least 18 tyrosine derivatives, including *p*-azido-L-phenylalanine (*p*AzF), *p*-acetylphenylalanine (*p*AcF), and so forth (Young et al. 2011). This polyspecificity has been applied to facilitate rapid and widespread use of the genetic ncAA incorporation technique in exploring the enzyme catalysis mechanism and engineering enzymes with improved properties. Homoserine *O*-succinyltransferase from *E. coli* was engineered to have improved stability with randomly incorporated ncAAs achieved by the polyspecific aminoacyl-tRNA synthetase/tRNA pair (Li et al. 2018). Also, three different ncAAs were incorporated into a transketolases variant from *E. coli* with the utility of *p*CNFRS to investigate the effect of aromatic ring electron density at a critical position on enzyme stability and catalysis efficiency (Wilkinson and Dalby 2021).

Similarly, both *Methanosarcina barkeri* pyrolysyl-tRNA synthetase (*Mb*PylRS) and *Methanosarcina mazei* pyrolysyl-tRNA synthetase (*Mm*PylRS) display wide substrate scope due to their high substrate side chain promiscuity and low selectivity toward the substrate α-amine. The native PylRS was demonstrated to accept more than 27 alternative substrates besides its native substrate pyrrolysine (Guo et al. 2014; Young and Schultz 2018). In addition, a rationally designed *Mm*PylRS variant (N346A/C348A) exhibited a wide substrate scope, which was able to incorporate over 40 phenylalanine (Phe) derivatives into proteins in *E. coli* and mammalian cells (Tuley et al. 2014; Tharp et al. 2014; Sharma et al. 2016). Phe is a large hydrophobic amino acid among 20 natural amino acids, aromatic ring of which forms hydrophobic interaction and π–π stacking interaction, critical to maintaining enzyme conformations and functions. Phe derivatives with functional groups different in size, polarity, and hydrophobicity could be potentially useful to expand the enzyme functions. In the *Mm*PylRS, N346 and C348 are hot spots for directed evolution of the enzyme to expand substrate scope (Wang et al. 2012, 2011; Guo et al. 2014; Englert et al. 2015). The amide nitrogen of the N346 side chain forms a hydrogen bond with the amide oxygen of the pyrrolysine (Pyl) side chain, anchoring Pyl in the active site (Wang et al. 2012). Mutation of C348 to an amino acid with a side chain that occupies the space of Pyl at the active site would bring more interactions with other ncAAs and hence modify the substrate specificity (Wang et al. 2011). Although N346A/C348A has been successfully used for the genetic incorporation of a variety of Phe derivatives (Tuley et al. 2014; Tharp et al. 2014; Sharma et al. 2016), for most of the substrates, the incorporation efficiency was low and there is a need to be further improved.

Like other PylRS in the archaeal genus *Methanosarcina*, *Mb*PylRS and *Mm*PylRS are organized in two conserved domains connected by a variable linker, including the tRNA-binding N-terminal domain (NTD) of around 120 residues and the C-terminal domain (CTD) of around 270 residues, which also binds tRNA and harbors the catalytic site (Suzuki et al. 2017). *Mb*PylRS and *Mm*PylRS possess a sequence identity of around 74%, but show different catalytic efficiency toward native substrate pyrrolysine, with 3.6-fold higher *k*_cat_/*K*_m_ in aminoacylation, and 3.1-fold lower *k*_cat_/*K*_m_ in amino acid activation for *Mb*PylRS compared to the *Mm*PylRS, respectively (Guo et al. 2014). When carrying the directed evolution of PylRS for incorporation of structurally diverse unnatural amino acids, mutations were commonly constructed in the CTD to expand the substrate scope. Although the effective mutations in CTD of PylRS from one species were also effective in another, it has been shown that the best mutation found in one species was not necessary the best in another (Koch et al. 2021). Mutations in the NTD have also been demonstrated to affect the ncAA incorporation efficiency of *Mm*PylRS, *Mb*PylRS variants, and their chimera (Suzuki et al. 2017). A *Mb*PylRS NTD variant containing six mutations (V8E, T13I, I36V, H45L, S121R, I355T) was reported to increase the incorporation of crotonyl lysine (Owens et al. 2017). These mutations could be transferred to other *Mb*PylRS variants containing mutations at C-terminal, leading to improved activity toward incorporation of corresponding ncAAs. A PylRS variant IPYE (V31I/T56P/H62Y/A100E) was obtained through a phage-assisted continuous evolution (PACE) selection, which showed improved activity and amino acid specificity. Transplantation of the evolved mutations into *Mb*PylRS and *Mm*PylRS variants greatly increased the activity as well (Bryson et al. 2017). In another study, a N-terminal mutation of *Mm*PylRS containing R19H/H29R/T122S was found to enhance the incorporation of ncAA and the yield of recombinant protein with the ncAA incorporated (Sharma et al. 2018). However, Williams et al. found that this mutation has negligible benefits when transferred to *Mb*PylRS (Williams et al. 2021).

In this study, we aimed to enhance ncAAs incorporation efficiency of a PylRS variant showing polysubstrate specificity, and apply it for exploring enzyme catalysis mechanism and engineering enzymes with improved properties. We first compared the activity of *Mb*PylRS(N311A/C313A) and *Mm*PylRS (N346A/C348A) against various substrates, and the better one, *Mb*PylRS (N311A/C313A), was chosen as a template for N-terminal mutation studies. We selected 10 N-terminal mutation sites from previous studies (Owens et al. 2017; Bryson et al. 2017; Neumann et al. 2008). To decrease the number of combined mutations from impractical all possible numbers to a controllable number, greedy optimization was used to evolve *Mb*PylRS(N311A/C313A) (Yu et al. 2022; Cui et al. 2021). After four rounds of greedy combination of single variants, highly active variant IPE (N311A/C313A/V31I/T56P/A100E) was obtained, and 16 novel ncAAs were then identified that could be incorporated into proteins using the IPE variant.

PedH is a lanthanide-dependent alcohol dehydrogenase that relies on lanthanide ions and pyrroloquinoline quinone (PQQ) as cofactors to oxidize a broad range of alcohol and aldehyde substrates (Keltjens et al. 2014; Wehrmann et al. 2017; Good et al. 2016; Jahn et al. 2018). Different from most oxidases, PedH has the advantage that no oxygen is required for catalytic reaction and no hydrogen peroxide is generated (Wehrmann et al. 2020). The best PylRS mutant was then used to individually incorporate 8 various ncAAs at substrate tunnel of the PedH. The enzyme properties of wild-type and mutant PedHs were characterized and found that incorporation of ncAAs altered the activity and enantioselectivity of PedH. It was also found that protein main-chain modification could also lead to the improvement in enzyme activity, potentially useful in future enzyme engineering.

## Results and discussion

### Active mutations transplant from *Mm*PylRS to *Mb*PylRS

**Fig. 1:**
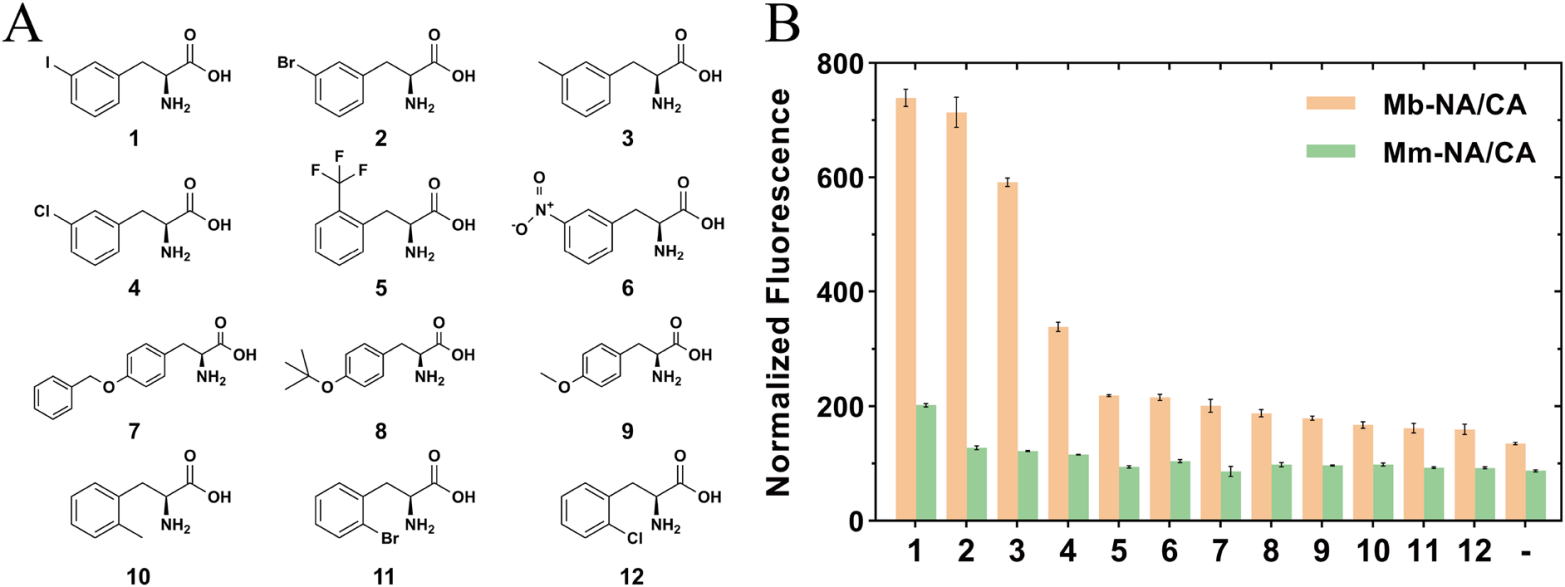
NcAA incorporation efficiency comparison between *Mm*PylRS (N346A/C348A) and *Mb*PylRS (N311A/C313A). **A** The chemical structures of 12 ncAAs used for testing incorporation efficiency. **1**, 3-iodo-L-Phe (3-I-Phe); **2**, 3-bromo-L-Phe (3-Br-Phe); **3**, 3-methyl-L-Phe (3-Me-Phe); **4**, 3-chloro-L-Phe (3-Cl-Phe); **5**, 2-trifluoromethyl-L-Phe (2-CF3-Phe); **6**, 3-nitro-L-Phe; **7**, *O*-benzyl-L-tyrosine; **8**, *O*-tert-butyl-L-tyrosine; **9**, *O*-methyl-L-tyrosine; **10**, 2-methyl-L-Phe (2-Me-Phe); **11**, 2-bromo-L-Phe (2-Br-Phe); **12**, 2-chloro-L-Phe (2-Cl-Phe). **B** Activity of Mm-NA/CA and Mb-NA/CA toward different ncAAs. Translation of the sfGFP reporter (UAG codon at position 2) by the two PylRS variants was measured by fluorescence intensity

*Mm*PylRS (N346A/C348A) has high substrate promiscuity and been reported to accept more than 40 ncAAs while maintaining relatively orthogonal toward canonical amino acids (Wan et al. 2014). It has been tested that *Mb*PylRS had a *k*_cat_/*K*_m_ value, around threefold higher than that of *Mm*PylRS in aminoacylation toward their native substrate Pyl (Suzuki et al. 2017). We hence wondered if the mutations in the *Mb*PylRS had higher incorporation efficiency toward different ncAAs compared to that in the *Mm*PylRS. The corresponding mutant N311A/C313A of *Mb*PylRS (Mb-NA/CA) was hence constructed and tested toward 12 different Phe analogs ncAAs for suppression efficiency with sfGFP assay (Fig. 1A). The sfGFP gene contained an in-frame amber stop codon at the position downstream of the first methionine encoding codon (sfGFP-R2TAG) and a C-terminal His-tag. In this way, the sfGFP fluorescence signal of intact cells can be used as readout for the in vivo suppression of the in-frame amber stop codon, with fluorescence intensity and full-length protein yields proportional to suppression efficiency. Expectedly, the fluorescence intensity of both *Mb*PylRS(N311A/C313A) and *Mm*PylRS(N346A/C348A) was above the background in the presence of the ncAAs, suggesting their success in activating the ncAAs (Fig. 1B). Also, the Mb-NA/CA showed higher fluorescence signals than Mm-NA/CA for all of the 12 ncAAs, indicating that the mutations in the *Mb*PylRS had higher efficiency in ncAAs activation and tRNA charging. Among the ncAAs tested, Mb-NA/CA showed significantly higher incorporation efficiency toward *meta*-substituted Phe derivatives, such as 3-iodo-L-Phe (**1**), 3-bromo-L-Phe (**2**), 3-methyl-L-Phe (**3**), and 3-chloro-L-Phe (**4**), compared to other ncAAs.

### Improvement of ncAA incorporation efficiency by N-terminal engineering

Mutations in the NTD of PylRS have been demonstrated to affect the efficiency of noncanonical amino acid incorporation. And, it was shown that modification of the NTD did not significantly influence the substrate specificity of the PylRS and could be transferred to different variants (Owens et al. 2017). We here attempted to test three sets of mutations obtained previously in the N-terminal of Mb-NA/CA to further improve its catalytic efficiency. One of them containing six mutations (EIVLRT, V8E, T13I, I36V, H45L, S121R, I355T) predominantly localized in the NTD were identified through random mutagenesis, which has a threefold increase in crotonyl lysine incorporation (Owens et al. 2017). Another one contained four mutations (IPYE, V31I, T56P, H62Y, A100E) that significantly improved the activity and amino acid specificity of PylRS (Bryson et al. 2017). The last one contained six mutations (D76G, L266V, L270I, Y271F, L274A, C313F) and was used to produce recombinant manganese superoxide dismutase bearing N^ε^-acetyllysine (Neumann et al. 2008). A total of 10 single-point mutations located at NTD were hence selected, including V8E, T13I, V31I, I36V, H45L, T56P, H62Y, D76G, A100E, and S121R for combination with the expectation to improve ncAA incorporation efficiency of Mb-NA/CA. However, due to the epistatic effect, these mutations may not cooperate to achieve the desired function. We hence used a greedy strategy to find the local optima (Cui et al. 2021). That is, starting from a single-point mutation, multiple rounds of iterative mutation were carried out, and only the mutants with the highest average incorporation efficiency for ncAAs were retained in each round until continued iteration could not improve the activity. Eight ncAAs (**1**, **2**, **3**, **5**, **8**, **9**, **11**, **12**) with different structures and properties were selected as substrates for testing.

**Fig. 2:**
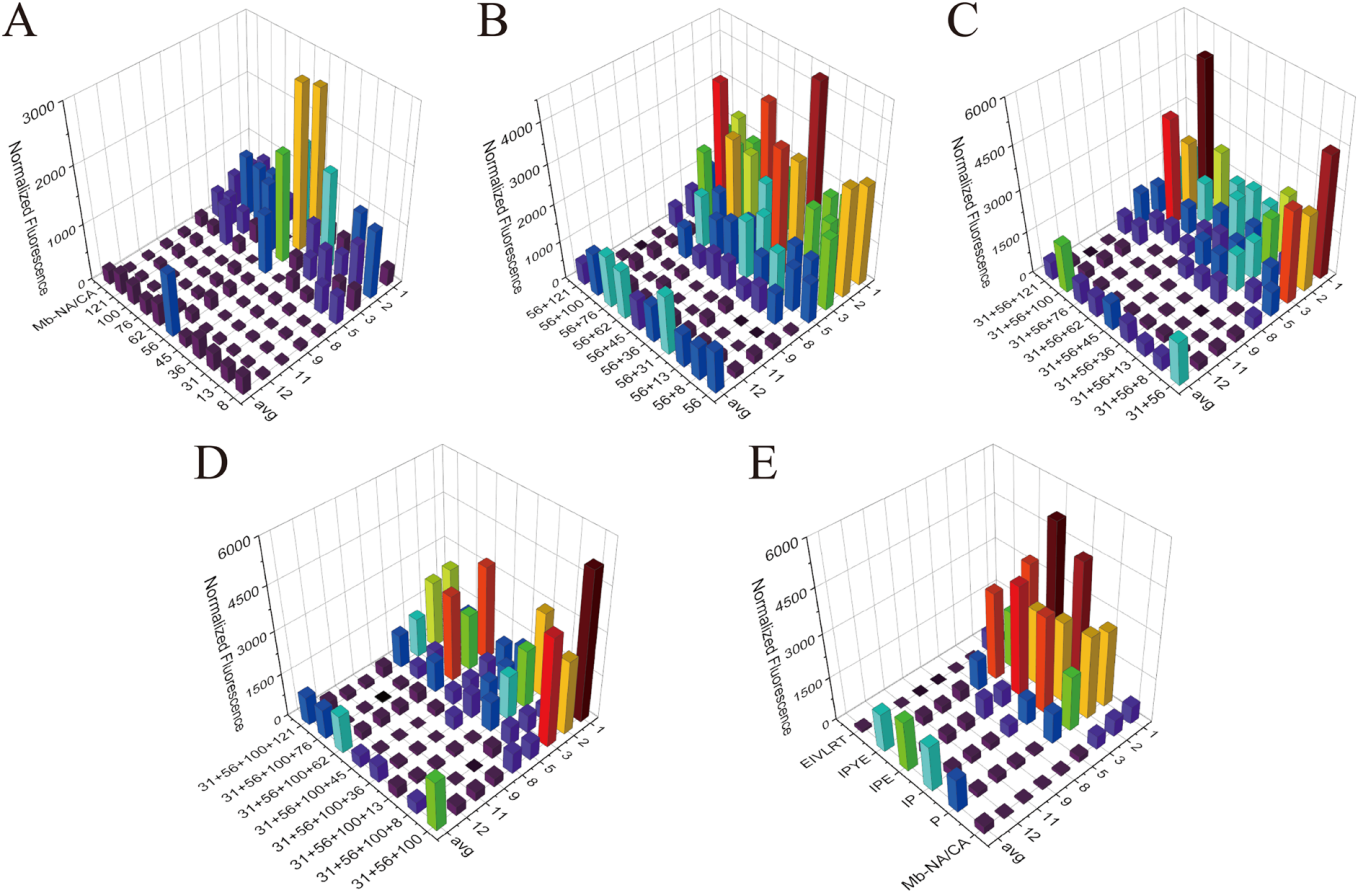
Incorporation efficiency of PylRS mutants toward 8 ncAAs during greedy evolution. **A** Incorporation efficiency of single mutants. Avg refers to the mutants’ average incorporation efficiency for the 8 ncAAs. **B** Incorporation efficiency of double mutants starting from T56P. **C** Incorporation efficiency of triple mutants starting from V31I/T56P. **D** Incorporation efficiency of quadruple mutants starting from V31I/T56P/A100E. **E** Comparison of incorporation efficiency between the optimal mutant obtained from each round of greedy evolution and IPYE, EIVLRT and Mb-NA/CA

Our data showed that 9 of 10 single mutants with the exception of H45L improved the incorporation efficiency toward at least one ncAA compared to the Mb-NA/CA. T56P performed best among the 10 single mutants in enhancing activity of Mb-NA/CA, with 5.2-fold, 4.9-fold, 4.3-fold improvement toward ncAAs **5**, **2**, and **1,** respectively (Fig. 2A). In order to obtain the PylRS variant showing high incorporation efficiency toward various ncAAs, the average incorporation efficiency of 8 different ncAAs was calculated, and that of T56P was increased by 3.4-times compared to Mb-NA/CA. H62Y had the second highest average incorporation efficiency, which was 1.6 times higher than Mb-NA/CA but only 36.0% of T56P. Furthermore, the average incorporation efficiency for different ncAAs of S121R and I36V were 1.5 and 1.4 times that of Mb-NA/CA, respectively.

Although the mutations are located at N-terminal, distantly from catalytic core of PylRS, they seemed to affect substrate specificity as well, as evidenced that the mutants improved activity of PylRS Mb-NA/CA against only specific ncAAs. For example, A100E significantly improved incorporation efficiency of ncAAs 3-Br-Phe (**2**) and 2-CF3-Phe (**5**) by 1.8-fold and 3.6-fold, respectively, whereas no huge positive effect was observed for other ncAAs. I36V only showed improved incorporation efficiency toward 3-Br-Phe (**2**). More interestingly, H62Y obviously increased incorporation efficiency of ncAAs 3-I-Phe (**1**) and 3-Me-Phe (**3**) by 2.6-fold and 2.7-fold, respectively, but dramatically decreased the suppression activity of Mb-NA/CA against ncAAs 3-Br-Phe (**2**) and 2-CF3-Phe (**5**) (Fig. 2A).

In the second round of greedy combination, V31I/T56P, T56P/H62Y, and T56P/D76G variants exhibited higher average incorporation efficiency toward 8 ncAAs than the T56P variant (Fig. 2B). Among the three great double variants, V31I/T56P showed the highest activity, 37.7% higher than the T56P. V31I/T56P was therefore selected as the template for the third round of greedy optimization and only one variant IPE (N311A/C313A/V31I/T56P/A100E) was found to have an average incorporation efficiency that exceeded the parent V31I/T56P (Fig. 2C). Whereafter, the greedy evolution was stopped since no variant with further enhanced activity was found in the fourth round (Fig. 2D). Our results demonstrated that the IPYE variant dramatically enhanced the activity of Mb-NA/CA, consistent with the previous observation that the mutation increased catalytic efficiency of both native and chimeric PylRS variants (Bryson et al. 2017). However, the optimal variant obtained in the four round greedy evolution was IPE, whose average ncAA incorporation efficiency was 29.9% higher than IPYE and 6.61-fold higher than that of Mb-NA/CA (Fig. 2E). This might be due to that the H62Y mutant affected the enzyme substrate selectivity and only be active toward some specific ncAAs. In addition, mutations EIVLRT showed negligible benefits when directly transferred to Mb-NA/CA (Fig. 2E), emphasizing that not all mutations at NTD were transferable between different MbPylRS CTD variants.

**Fig. 3:**
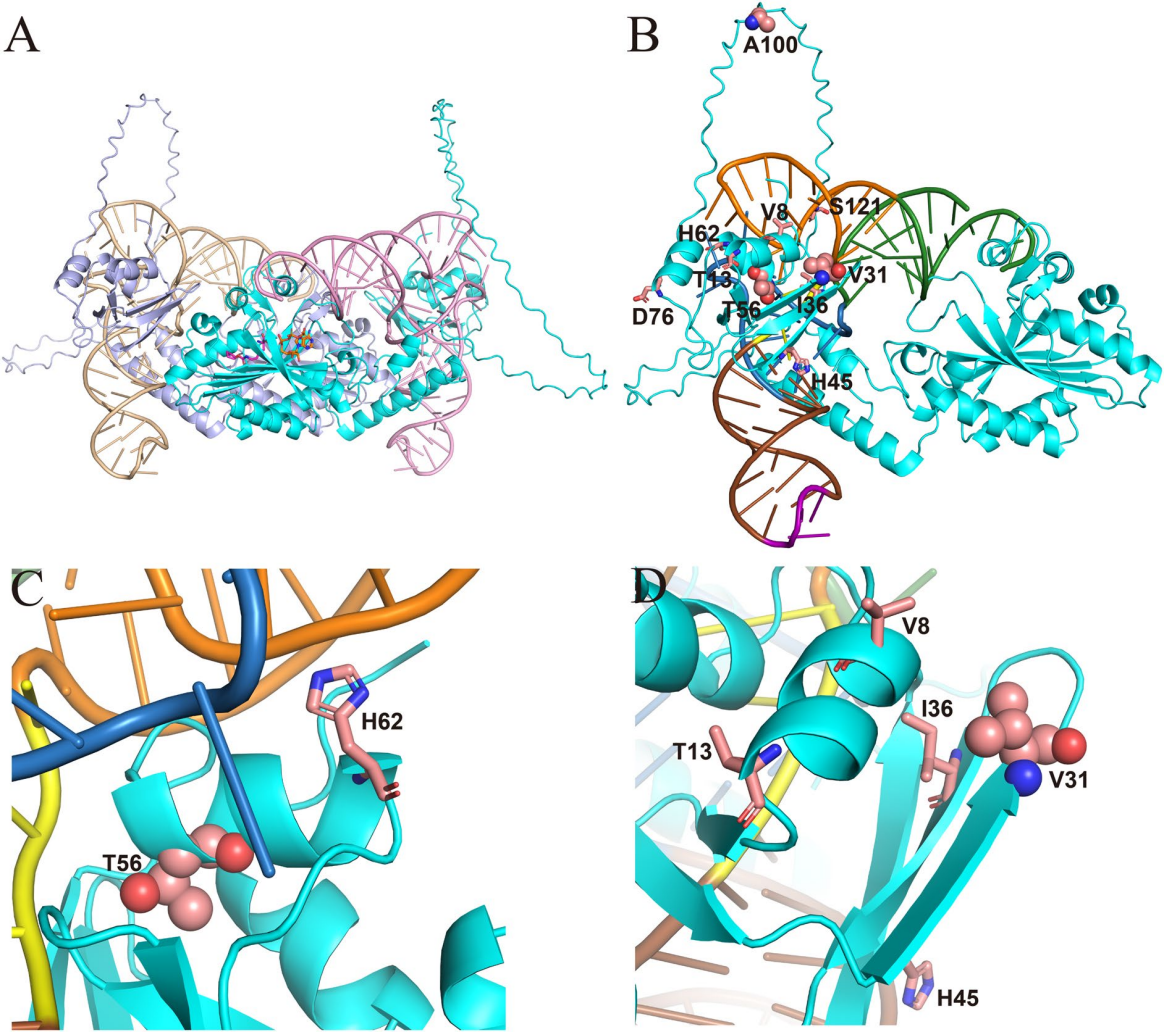
Analysis of *Mb*PylRS complex structure bound with tRNA^Pyl^. **A** Superposition of the *Mb*PylRS-tRNA^Pyl^ complex predicted by AlphaFold2. **B** N-terminal residues selected for greedy evolution are represented as sticks, with V31, T56 and A100 represented as spheres. The secondary structure features of tRNA^Pyl^ are colored: blue, D loop; brown, anticodon stem; purple, anticodon; yellow, variable loop; orange, T loop; green, acceptor stem. **C** Close-up view of T56 and the surrounding residues. **D** Close-up view of V31 and the surrounding residues

The complete crystal structures of *Mb*PylRS and the *Mb*PylRS-tRNA^Pyl^ complex have not yet been determined. In order to better understand the molecular mechanism of how the mutations in NTD affect the incorporation efficiency of ncAAs, we used Alphafold2-multimer to predict the dimeric structure of *Mb*PylRS. The predicted local distance difference test (plDDT) scores of the five structures generated by Alphafold2-multimer are shown in Additional file 1: Fig. S1. As expected, the model confidence of the variable linker between NTD and CTD was low in all five structures. The rank_1 model with the highest plDDT score was selected for further analysis (Additional file 1: Fig. S2), and the model without the variable linker was submitted to the SAVES v6.0 server for quality evaluation using four different methods, including Errat, Verify3D, Whatcheck, and Procheck. The structure was found to pass the checks of these methods and showed a relatively high accuracy (Additional file 1: Fig. S3). Due to the high sequence identity (74%) between *Mb*PylRS and *Mm*PylRS, and the similarity between their homologous tRNA^Pyl^ (Additional file 1: Fig. S4, S5), the rank_1 model was superimposed with the *Mm*PylRS NTD-tRNA^Pyl^ complex (PDB: 5UD5) and the *Mm*PylRS CTD-ATP-*N*-ε-[(cyclopentyloxy)carbonyl]-l-lysine (Cyc) complex (PDB: 2Q7G) to obtain the *Mb*PylRS-tRNA^Pyl^-ATP-Cyc complex structure (Fig. 3A) (Suzuki et al. 2017; Kavran et al. 2007; Tharp et al. 2018). The wild-type NTD was shown in Fig. 3B, with 10 selected single-point mutation sites highlighted. Previous study showed that the H62Y mutation disrupts two hydrogen bonds between the *Mm*PylRS and T loop moieties of tRNA^Pyl^, but it establishes new, and seemingly weaker, contacts with a phosphate moiety of G21 and the base of A20 (Suzuki et al. 2017; Jiang et al. 2020). The reconstructed interactions might impact the enzyme substrate specificity through the tRNA as a bridge. Given the distinctive cyclic R-group of proline, the T56P mutation would disrupt the α-helix at this site, consequently impacting the interaction between *Mb*PylRS NTD and tRNA^Pyl^ (Fig. 3C). V31, I36, and H45 are located at adjacent β-sheets in close proximity to the α-helix containing V8 and T13. Mutations at these positions may potentially alter the interactions between the β-sheets and the α-helix. Disturbance in the secondary structures of *Mb*PylRS NTD could affect its binding with tRNA^Pyl^ (Fig. 3D). E100 and S121 are located in the variable linker region of *Mb*PylRS, and in the actual catalysis process of *Mb*PylRS-tRNA^Pyl^, these sites may interact with the T loop or acceptor stem of tRNA^Pyl^, instead of being away from tRNA^Pyl^ as shown in the predicted structure (Fig. 3B).

### N-terminal engineering of pyrrolysyl-tRNA synthetase enables incorporation of 16 novel noncanonical amino acids

**Fig. 4:**
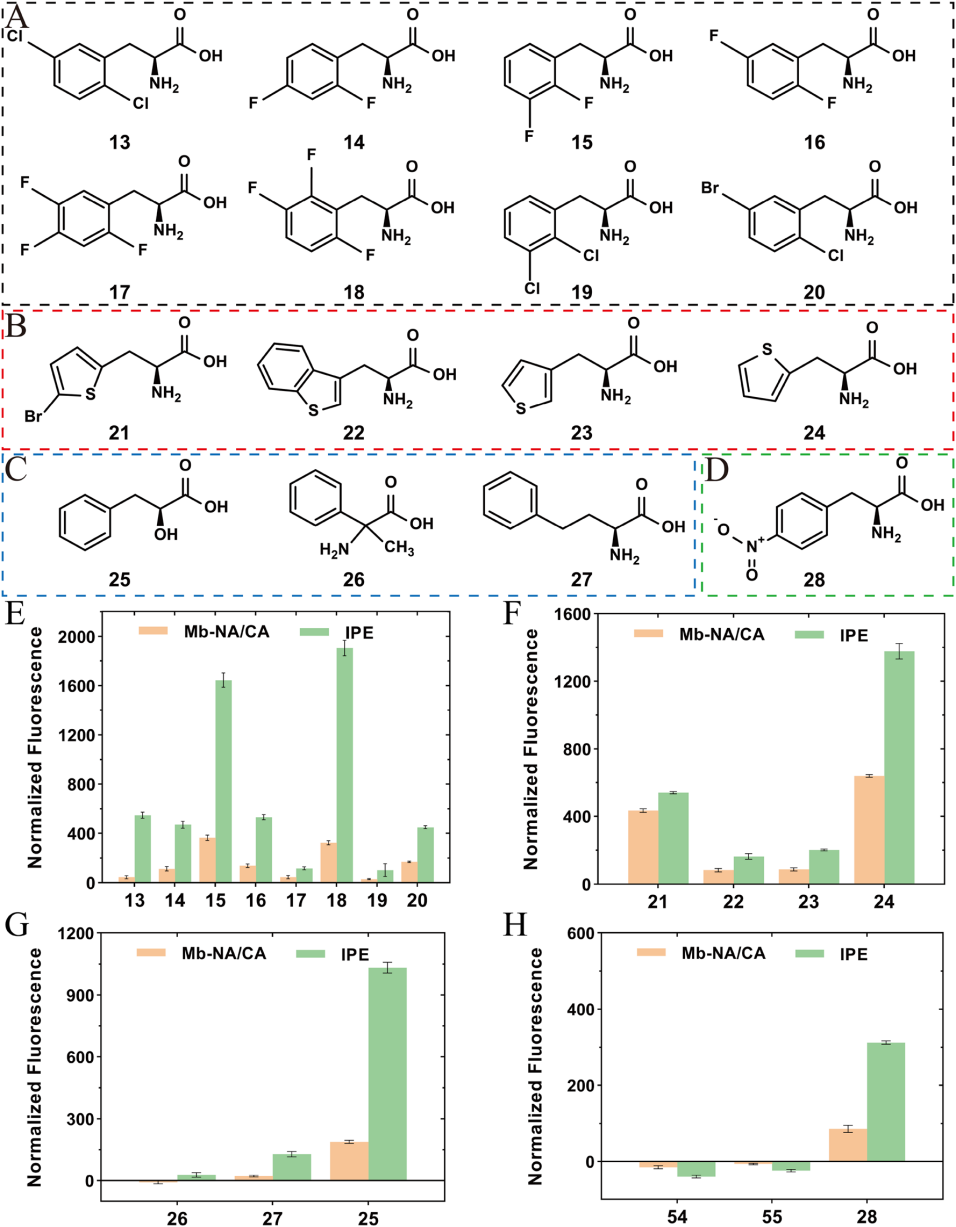
Molecular structures of the 16 novel ncAAs in four classes accepted by IPE variant and the incorporation efficiency of Mb-NA/CA and IPE toward these ncAAs. Structures of **A** halogenated Phes, **B** thienyl-L-alanines, **C** non-phenyl-substituted Phes, **D** *para*-substituted Phes. Incorporation efficiency of Mb-NA/CA and IPE toward **E** halogenated Phes, **F** thienyl-L-alanines, **G** non-phenyl-substituted Phes, **H** *para*-substituted Phes. **13**, L-2,5-dichloroPhe; **14**, L-2,4-difluoroPhe; **15**, L-2,3-difluoroPhe; **16**, L-2,5-difluoroPhe; **17**, L-2,4,5-trifluoroPhe; **18**, L-2,3,6-trifluoroPhe; **19**, L-2,3-dichloroPhe; **20**, 5-bromo-2-chloro-L-Phe; **21**, L-2-(5-bromothienyl)alanine; **22**, L-3-benzothienylalanine; **23**, 3-(3-thienyl)-L-alanine; **24**, 3-(2-thienyl)-L-alanine; **25**, Phenyl-lactic acid; **26**, 2-amino-2-phenylpropionic acid; **27**, L-homoPhe; **28**, 4-nitro-L-Phe

PylRS variant NA/CA is promiscuous and more than 40 ncAAs could be incorporated into proteins with utility of this variant. To test whether the engineering of NTD of PylRS promotes the incorporation of other ncAAs, we measured activity of the IPE variant toward 43 ncAAs that have not been reported to be recognized by Mm-NA/CA or Mb-NA/CA (Additional file 1: Fig. S6). These ncAAs include Phe derivatives, cysteine derivatives, leucine derivatives, tryptophan derivatives, lysine derivatives, etc. Surprisingly, compared with Mb-NA/CA, the IPE variant showed significantly increased incorporation efficiency toward 16 of them, which could be classified into four categories according to their properties, including halogenated Phes, thienyl-L-alanines, nonphenyl-substituted Phes and *para*-substituted Phes (Fig. 4A–D). This reflected that N-terminal engineering mutation of PylRS variants was able to improve incorporation efficiency of a wide range of ncAAs. Furthermore, these four categories of noncanonical amino acids were incorporated into sfGFP and verified by protein purification, SDS-PAGE and LC–MS analysis (Additional file 1: Fig. S7 and Table 1). The deconvoluted ESI–MS spectrum of the purified full-length sfGFP proteins is shown in Additional file 1: Fig. S8.

**Table 1:** Mass spectra to investigate incorporation of 16 ncAAs by Mb-NA/CA-IPE

ncAA	Expected^a^ (Da)	Observed (Da)
13	27856.89	27857.38
14	27823.98	27788.42, 27826.84
15	27823.98	27824.63
16	27823.98	27788.46, 27825.32
17	27841.97	27842.54
18	27841.97	27842.36
19	27856.89	27856.99
20	27901.34	27901.85
21	27872.92	27873.39
22	27844.09	27844.63
23	27794.03	27792.26
24	27794.03	27790.66
25	27788.98	27788.49
26	27788.0	27788.81
27	27802.03	27769.88, 27788.70, 27805.78
28	27833.0	27788.76, 27831.27

^a^LC–MS expected mass of sfGFP-S2TAG with ncAAs incorporated

#### Halogenated Phes

The IPE showed significantly improved UAG translation against halogenated Phes compared to the Mb-NA/CA, with the largest 4.9-fold improvement for L-2,3,6-trifluoroPhe (**18**) (Fig. 4E). Although L-2,4,5-trifluoroPhe (**17**) and L-2,3-dichloroPhe (**19**) exhibited relatively lower incorporation efficiency compared to other ncAAs (Fig. 4E–H), they were indeed incorporated into sfGFP by IPE as evidenced by the SDS-PAGE and mass spectra (Additional file 1: Fig. S7, S8 and Table 1). It was interesting to find that both the Mb-NA/CA and IPE were sensitive to the halogen group substitution position in the phenyl ring of Phe. For example, Phe analogs (**13** and **20**) showed same substitution position and the IPE exhibited similar incorporation efficiency for them. DifluoroPhes (**14, 15, 16**) clearly showed different incorporation efficiency due to the different fluoro substitution position at the phenyl ring, similar with the observation of trifluoroPhes (**17, 18**). Among them, mass spectra revealed that the IPE variant also incorporated native Phe when incorporating **14** or **16** into sfGFP, as the mass of sfGFP with Phe incorporated is exactly 27788 Da (Table 1 and Additional file 1: Fig. S8). Since Mm-NA/CA has been proved to successfully incorporate fluoroPhes including pentafluoro-Phe, L-2,3,4,5-tetrafluoroPhe, L-3,4,5-trifluoroPhe, L-3,5-difluoroPhe, and L-3,4-difluoroPhe, it was postulated that IPE variant would also improve the incorporation efficiency toward those five fluoroPhes based on the results of fluoroPhes (**14–18**). The genetic installation of these halogenated Phes in proteins would provide multiple ways to site-selectively label proteins with biophysical and biochemical probes for their functional investigations (Brittain et al. 2020). For instance, ncAAs with fluorine atoms can serve as a sensitive ^19^F NMR probe for protein folding/unfolding and structural dynamics analysis (Cobb and Murphy 2009). Perfluoroaromatic moieties can have significant effects on the electronics of molecular systems due to their highly electron deficient nature (Fuhrer et al. 2019).

##### Thienyl-L-alanines

**Fig. 5:**
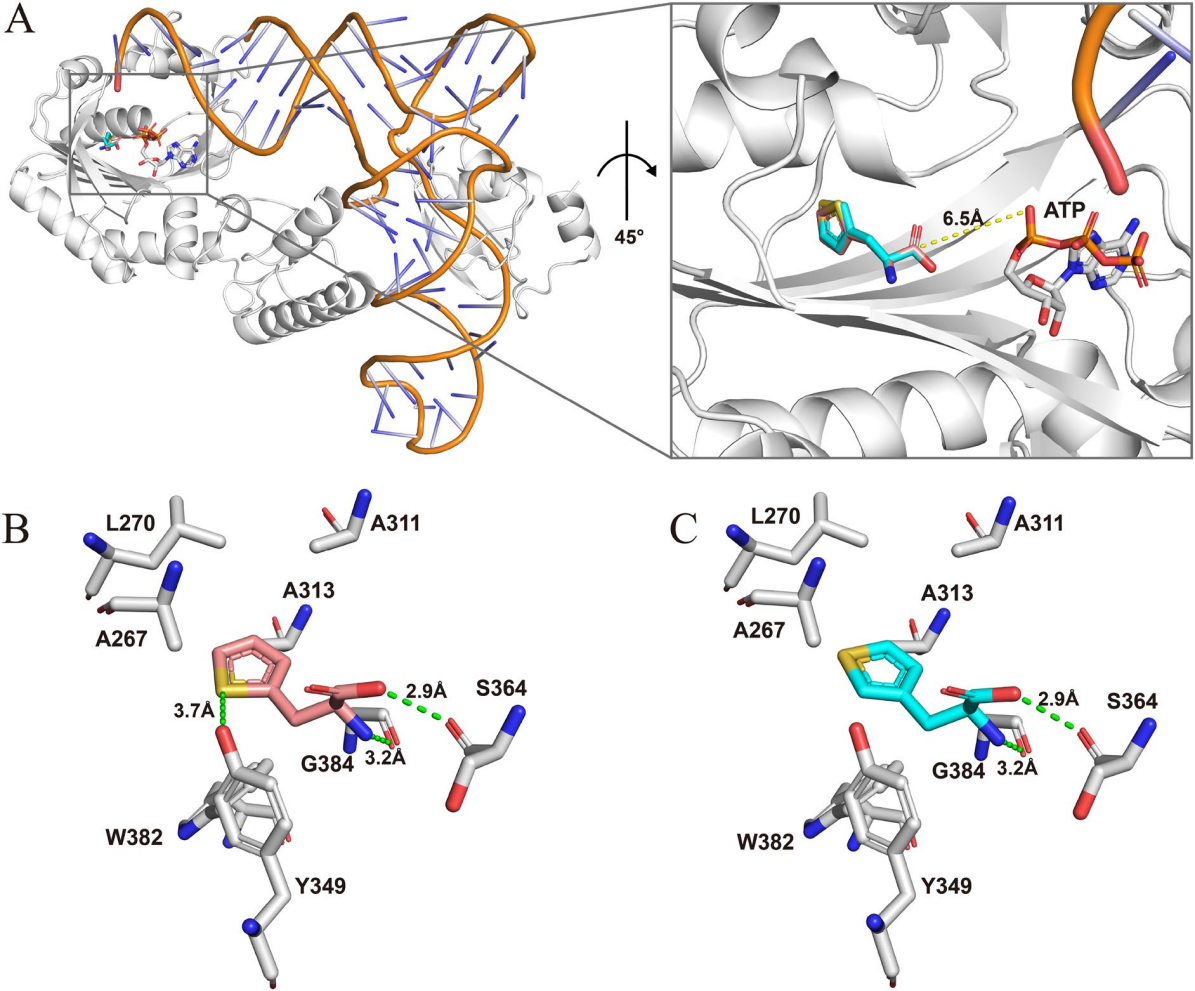
Structural features of the **23**/**24**-IPE-binding sites and the key residues. **A** Superposition of the binding conformations of **23** and **24** with the IPE-tRNA^Pyl^ monomer. 3-(3-thienyl)-L-alanine (**23**) is represented as cyan sticks, and 3-(2-thienyl)-L-alanine (**24**) is represented as salmon sticks. **B** Interaction between **24** and the surrounding residues. **C** Interaction between **23** and the surrounding residues

L-3-benzothienylalanine (Bta**, 22**) is an analog of tryptophan and has been successfully incorporated into proteins with *Mm*PylRS variant N346G/C348Q (Englert et al. 2015). Bta possesses an imino-to-sulfur substitution in the five-membered ring of tryptophan and has been applied to investigate catalytic mechanism of thioredoxin. We here showed that Mb-NA/CA could also incorporate this ncAA into sfGFP and the IPE variant further improved its incorporation efficiency by 2.0-fold (Fig. 4F and Table 1). Similarly, the IPE variant improved the incorporation efficiency of other three similar ncAAs, with the largest improvement for 3-(2-thienyl)-L-alanine (**24**), 2.2-fold higher than that of Mb-NA/CA. Interestingly, 3-(3-thienyl)-L-alanine (**23**) showed lower incorporation efficiency compared to 3-(2-thienyl)-L-alanine (**24**)**,** although they only differ in the sulfur substitution position in the five-membered ring, suggesting the role of substitution position in PylRS catalytic efficiency. To understand how the different substituent positions affect the catalytic efficiency of PylRS, we performed molecular docking of **23** and **24** with the CTD of IPE variant and similar conformations were observed for **23** and **24** in the *Mb*PylRS active center (Fig. 5A). Both **23** and **24** were surrounded by a hydrophobic pocket formed by residues A267, L270, A311, A313, and W382, and both of them formed hydrogen bonds with the backbone oxygen atoms of S364 and G384 (Fig. 5B, C). However, a notable difference was observed that the sulfur atom on the five-membered ring of **24** formed a hydrogen bond with the hydroxyl group of Y349, but **23** could not form this hydrogen bond due to the change in the position of the sulfur substituent. This may cause the binding affinity of **23** to be lower than that of **24**, which explains why the incorporation efficiency of **23** by IPE and *Mb*PylRS is significantly lower than that of **24**. When the docking results were superimposed with the *Mm*PylRS CTD-ATP-Cyc complex (PDB: 2Q7G), it was found that the five-membered ring of 3-(3-thienyl)-L-alanine (**23**) and 3-(2-thienyl)-L-alanine (**24**) showed a certain of deviation from the five-membered ring of Cyc (Additional file 1: Fig. S9). Unlike Cyc being adjacent to ATP, the carboxyl carbons of **23** and **24** are about 6.5 Å away from the closest hydroxyl group of ATP, indicating that no reaction will occur at this protein conformation (Additional file 1: Fig. S9). Therefore, we speculate that **23** and **24** may first bind with CTD in an inactive form and then transition to the active-form position for reaction with ATP, similar to a previous study (Vatansever et al. 2022).

##### Nonphenyl-substituted Phes

PylRS has been proved to recognize substrates with α-substituents different from amine, and α-hydroxy acids analogs of lysine or Phe could be incorporated into proteins with evolved PylRS-tRNA^pyl^ pair (Wan et al. 2014; Spinck et al. 2023). Here, as shown in Fig. 4G, the activity of IPE was 5.5 times higher than that of Mb-NA/CA toward phenyllactic acid (**25**), a α-hydroxy acid analog of phenylalanine. When the phenyllactic acid **25** is incorporated into a protein, it will form a relatively weak ester bond and the oxygen atom of the main chain can solely function as a hydrogen bond acceptor. The enzyme reaction is generally catalyzed through the side chains of key amino acid residues, and protein engineering is also aimed at modification of the side chains of amino acids. The incorporation of phenyllactic acid in protein may provide a tool to probe the effect of main chain nitrogen atom on enzyme stability and activity. Compared to Mb-NA/CA, the IPE variant also appeared to weakly improve the efficiency of incorporating 2-amino-2-phenylpropionic acid (**26**) and L-homoPhe (**27**) (Fig. 4G and Additional file 1: Fig. S7). LC–MS analysis revealed that the mass of sfGFP produced by IPE (**26**) was 27788.81 Da, which corresponded to the expected mass of 27788 Da (Table 1 and Additional file 1: Fig. S8). However, this did not rule out the possibility of Phe misincorporation, as the mass of sfGFP with Phe misincorporated was also found to be 27788 Da. The mass of sfGFP produced by IPE (**27**) was found to have three different values: 27769.88 Da, 27788.70 Da and 27805.78 Da, among which 27805.78 Da was consistent with the expected mass of 27802 Da (Table 1 and Additional file 1: Fig. S8). Analysis of SDS-PAGE revealed that the sfGFP protein bands produced by IPE in presence of **26** and **27** were more prominent than in absence of these two ncAAs, indicating that IPE was indeed able to incorporate **26** and **27** into a protein (Additional file 1: Fig. S7). Furthermore, it was found that IPE would misincorporate Lys and Phe when the activities of the ncAAs added in the media were not high, as evidenced by the peaks of 27769 Da and 27,788 Da corresponding to incorporation of Lys and Phe in the sfGFP.

##### *Para*-substituted Phes

This group of ncAAs comprises derivatives of Phe, and the substituents are in the *para* position of the phenyl ring. Neither Mb-NA/CA nor IPE was able to incorporate 4-amino-L-Phe (**54**) or L-4-cyano-L-Phe (**55**) into sfGFP (Fig. 4H). However, both of them exhibited low activity toward 4-nitro-L-Phe (**28**) (Fig. 4H and S7). The expected mass of sfGFP incorporated with 4-nitro-L-Phe (**28**) is 27833.0 Da, consistent with the mass observed in LC–MS analysis (27831.27 Da). Nonetheless, the presence of another mass (27788.76 Da) indicated that the majority of incorporation in the protein is Phe, with only a limited-portion incorporation of **28** (Table 1 and Additional file 1: Fig. S8). Studies on *Mm*PylRS revealed that the N346A mutation not only significantly decreased the binding of PylRS with Pyl, but also relieved the steric hindrance that prevents the binding of Phe (Wang et al. 2012; Kurra et al. 2014). Meanwhile, the C348A mutation was thought to generate a larger hydrophobic active site pocket, making it easier to recognize Phe derivatives with bulky and hydrophobic *para* substituents instead of smaller ones. This was consistent with the observation that Mb-NA/CA and IPE variant were active against **7**, **8**, and **9** possessing large *para* substituents, but inactive against 4-fluoro-L-Phe (**29**) with small *para* substituents. The steric hindrance of the *para* substituents of **28**, **54**, and **55** was similar to *O*-methyl-L-tyrosine (**9**), but the IPE variant had low or no activity against them. This may have resulted in by the polarity of the substituents of **28**, **54**, and **55** that was unfavorable for binding the hydrophobic substrate pocket, which led to the difficulty in recognition by the IPE variant. So far, various *para*-substituted Phes have been successfully incorporated into proteins. These ncAAs are expected to be used to identify substrate channels, explore enzyme stereoselectivity mechanisms or perturb enzyme catalysis further away from the main chain.

### Probing the role of a conserved Phe412 in an alcohol dehydrogenase

**Fig. 6:**
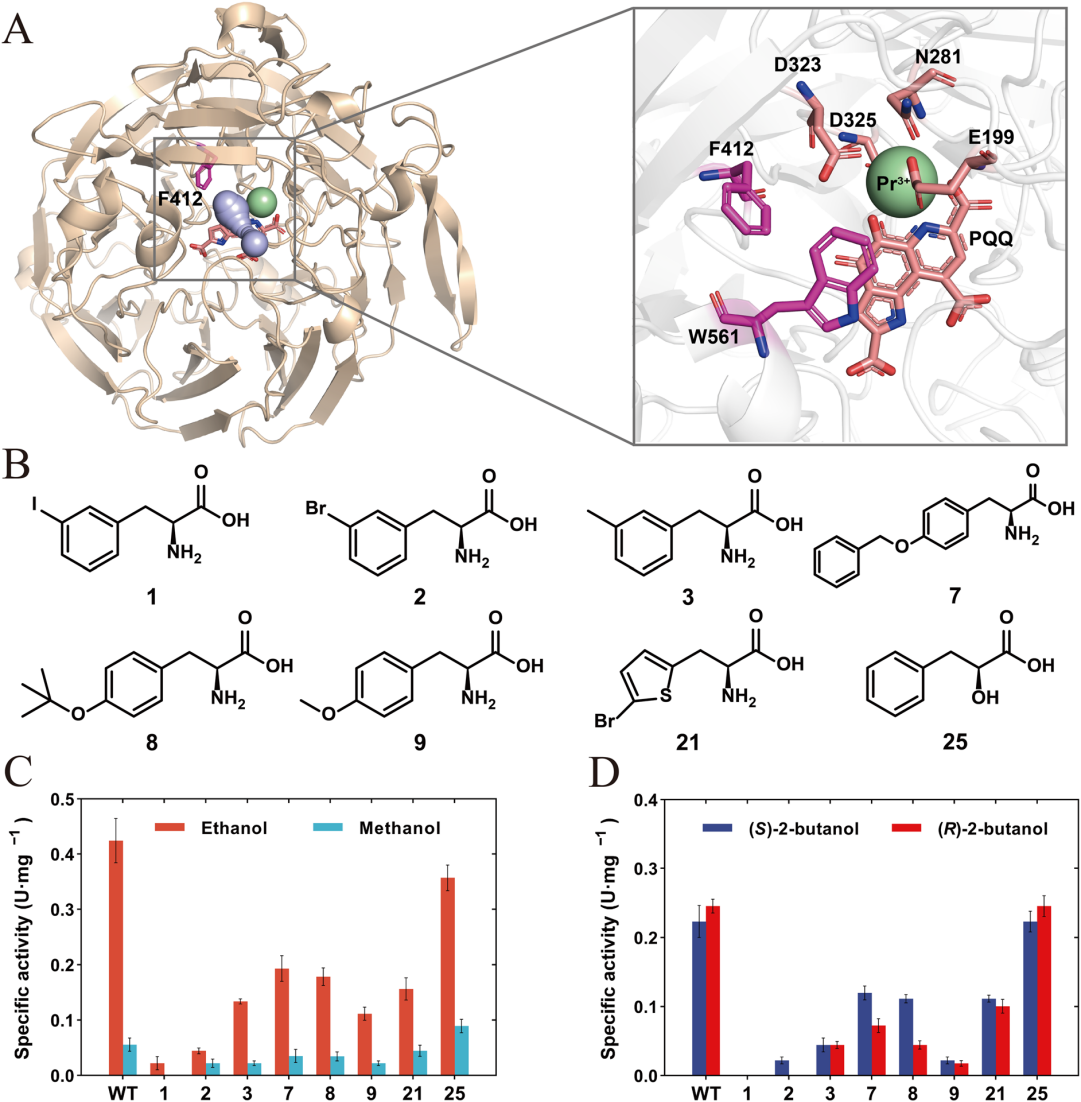
Incorporation of 8 ncAAs into the 412 site of PedH. **A** Crystal structure of PedH (PDB ID 6ZCW) and close-up view around the 412 site of PedH. The light blue channel indicates the substrate channel predicted by Caver 3. **B** Structures of the 8 ncAAs incorporated into PedH. **C** Specific activities of the variants toward ethanol and methanol. **D** Specific activities of the variants toward (*S*)-2-butanol and (*R*)-2-butanol

PylRS IPE variant is now potentially able to incorporate nearly 60 ncAAs including more than 40 ones determined previously (Wan et al. 2014), and 16 ones identified above into proteins, which provides a valuable platform for illustrating enzyme catalysis mechanism and modifying enzyme structures and functions. In order to illustrate a possible application of the PylRS IPE variant, we used it to incorporate 8 ncAAs with various structures and properties into a lanthanide-dependent alcohol dehydrogenase from *Pseudomonas putida* KT2440 (PedH) (Fig. 6). Lanthanide-dependent methanol dehydrogenase was discovered in 2011 in methylotrophic bacteria, marking the first known instance of the rare-earth elements in a physiological role. PedH was the solely Ln-dependent alcohol dehydrogenase known to be successfully expressed in *E. coli* without addition of any protein tags. Increasing the understanding of relationship between structure and function of PedH is useful to decode the physiological role of rare-earth element and expand applications of the enzyme in industry. In the present study, Caver 3 was utilized to predict the substrate channel of PedH and found a critical F412 located at the substrate channel (Fig. 6A). An enzyme engineering study showed that mutations at this position increased activity toward non-native substrates (Wehrmann et al. 2020). We hence chose to incorporate 8 Phe derivatives into the F412 site of PedH to investigate their perturbation to activity and enantioselectivity (Fig. 6B).

Wild-type and mutant PedHs were purified and detected with SDS-PAGE, showing the successful incorporation of the 8 ncAAs (Additional file 1: Fig. S10). The enzyme activity was then tested against different substrates including methanol, ethanol, (*S*)-2-butanol and (*R*)-2-butanol (Fig. 6C, D). Compared to the wild-type protein, most mutants showed a decrease in activity toward the substrates tested. Among them, the mutants with the incorporation of Phe derivatives 3-I-Phe (**1**), 3-Br-Phe (**2**), and 3-Me-Phe (**3**) having *meta*-substituents showed the most significant reduction in activity. The activity of the three PedH mutants with *meta*-substituted groups of **3**, **2**, **1** decreased in order as the steric hindrance of the substituent increased, indicating that the steric hindrance of the *meta*-substituted group may directly affect the substrate binding (Additional file 1: Fig. S11). Moreover, the incorporation of *O*-benzyl-L-tyrosine (**7**) and *O*-tert-Butyl-L-tyrosine (**8**) changed the enantioselectivity of PedH which showed higher activity toward (*S*)-2-butanol than (*R*)-2-butanol, while the wild type exhibited similar activity against the two substrates. Kinetic parameters of wild type and variant F412OBT exhibited that the variant decreased the *K*_m_ values against (*S*)-2-butanol and (*R*)-2-butanol compared to the wild type (Additional file 1: Table S1). MD simulations showed that due to the incorporation of **8**, oxygen of (*S*)-2-butanol was found to exhibit a shorter distance with C5 of PQQ compared to the (*R*)-2-butanol (Additional file 1: Fig. S12 and S13). Based on the reaction catalysis mechanism, the shorter distance might contribute to the enhanced activity, which might explain the higher activity of the variant toward (*S*)-2-butanol than (*R*)-2-butanol.

The incorporation of phenyl-lactic acid (**25**) surprisingly increased the specific activity of PedH toward methanol by 1.6-fold, from 0.056 U/mg to 0.089 U/mg, although it slightly reduced the specific activity toward ethanol (Fig. 6C). Phenyl-lactic acid (**25**) forms an ester bond with other amino acids instead of the peptide bond. In general, the main chain of natural enzyme is connected by peptide bonds, forming the backbone of protein and basically does not participate in catalytic reaction. Since enzyme catalysis is mainly performed through the side chains of key amino acid residues, few studies have investigated the effect of modifying main chain atoms on the catalytic properties of enzymes. In order to better understand the effect of phenyl-lactic acid (**25**) on enzyme catalysis, kinetic parameters of F412PLA variant with phenyl-lactic acid incorporated against the substrate methanol were determined (Additional file 1: Table S1). Compared with the wild type, the *K*_m_ value of F412PLA decreased from 1.10 mM to 0.70 mM, and the *k*_cat_ value increased from 0.15 s^−1^ to 0.17 s^−1^, which led to a 1.8-fold improved *k*_cat_/*K*_m_ for the variant compared to the wild type, indicating that the subtle transition from peptide bond to ester bond in the main chain indeed improved the catalytic performance of the mutant toward methanol.

The interaction between site F412 and surrounding residues was explored to investigate the impact of this subtle transition. MD simulations revealed that a hydrogen bond existed between the sulfur atom of M439 and the nitrogen atom on the main chain of F412 (Additional file 1: Fig. S14). The introduction of a phenyl-lactic acid disrupted the hydrogen bond, which perturbed the hydrogen bond interaction network and dynamics of active sites, thereby affecting enzyme activity (Additional file 1: Fig. S14, S15). These results indicated that subtle modification of a critical residue F412 by removing its main-chain hydrogen bond resulted in a change to the residue conformation, which enhanced the activity toward the small-sized substrate methanol, but not the large-sized substrate ethanol, exhibiting the importance of modifying protein main-chain atoms in engineering enzyme substrate specificity.

## Conclusion

In conclusion, *Mb*PylRS(N311A/C313A) was found to have a higher incorporation efficiency of various ncAAs compared to *Mm*PylRS(N346A/C348A). And the N-terminal engineering of *Mb*PylRS(N311A/C313A) resulted in a variant IPE, expanding the substrate scope of *Mm*PylRS(N346A/C348A) to nearly 60 ncAAs. With the utility of the variant, eight various ncAAs were incorporated into PedH, and it was found that the incorporation of phenyl-lactic acid improved the catalytic efficiency toward methanol by 1.8-fold compared to the wild type, and the incorporation of *O*-tert-Butyl-L-tyrosine mediated the enantioselectivity of PedH. Enzymatic characterization and MD simulations showed that incorporation of ncAAs could significantly or subtly modify the interactions between substrate and surrounding residues. This work increased the utility of orthogonal translation systems and indicated that protein main-chain modification, enabled by the incorporation of ncAA, might provide a new approach for protein engineering.

## Materials and methods

### Microorganisms and materials

*Mb*PylRS, *Mm*PylRS, cognate tRNA^Pyl^, sfGFP-S2TAG reporter, and PedH used in this work are preserved in the laboratory and expressed in the host *E. coli* BL21(DE3). The expression vectors used in this study were pBK, pULTRA, and pET-28a. The ncAAs were purchased from Bide Pharmatech Ltd. (Shanghai, China). Methanol and ethanol were provided by Sinopharm Chemical Reagent Co., Ltd. (*S*)-2-butanol and (*R*)-2-butanol were obtained from Shanghai Macklin Biochemical Co., Ltd. DNA polymerase (PrimeStar Max) was purchased from Takara Biomedical Technology (Beijing) Co., Ltd.

### Construction of PylRS mutants

*Mb*PylRS variant and *Mm*PylRS were cloned between the NdeI site and PstI sites of pBK-PylS plasmid (a kind gift from Dr. Jason Chin, Medical Research Council Laboratory of Molecular Biology) (Neumann et al. 2008). Mb-NA/CA, Mm-NA/CA, and other N-terminal mutations were introduced into each PylRS by PCR using primers in Additional file 1: Table S2.

### PylRS activity measurement by the sfGFP assay

12 ncAAs were selected for the purpose of testing PylRS activity. The sfGFP-S2TAG reporter with cognate tRNA^Pyl^ from *Methanosarcina barkeri* and the pBK-PylRS variants plasmids were transformed into *E. coli* BL21 (DE3). The stains were cultured in LB media at 37°C, supplemented with tetracycline (12.5 μg/mL), kanamycin (25 μg/mL). After 6–8 h, cells were harvested by centrifugation at 5,000 × g for 10 min, and then re-suspended in GMML media. Cells were cultured reaching to an OD_600_ of 0.2, and transferred to a clear bottom 96-well plate. At the same time, L-arabinose (5 mg/ml) and various ncAAs (1 mM) were also added separately. After culturing for 16 h at 30°C, the GFP fluorescence was measured using a plate reader with 485 nm excitation and 515 nm emission.

### Expression and purification of sfGFP and variants

The *Mb*PylRS variant which contained five mutations (N311A/C313A/V31I/T56P/A100E) was cloned into pBK vector and named pBK-PylRS-IPE. The sfGFP-S2TAG reporter with cognate tRNA^Pyl^ and the pBK-PylRS-IPE were transformed into *E. coli* DH10B. Cells were added into LB media (10 ml) containing tetracycline (12.5 μg/mL) and kanamycin (25 μg/mL), cultured at 37°C overnight and diluted into LB media (400 mL) to be incubated at 37°C until OD_600_ = 0.4–0.6. L-arabinose was added to reach the final concentration of 5 mg/mL, and non-canonical amino acid was added to reach the final concentration of 1 mM. Cultures were incubated at 30°C for 16 h with constant agitation for protein production. Protein was purified using Ni–NTA resin. The protein size was verified by SDS-PAGE.

### Expression and purification of PedH and variants

The nucleic acid sequence of PedH was placed in plasmid pET28a and was mutated to an amber codon at the 412 site named pET-PedH-F412TAG. The primers used here are shown in Table S3. The *Mb*PylRS (N311A/C313A/IPE) was cloned into a pULTRA vector, a gift from Peter Schultz (Addgene plasmid #48215) to generate plasmid pULTRA-PylRS-IPE. Plasmid pULTRA-PylRS-IPE was co-transformed into *E. coli* BL21 (DE3) with pET-PedH-F412TAG. Cells were added into LB media (10 mL) containing kanamycin (50 µg/mL) and spectinomycin (50 µg/mL), cultured at 37°C overnight and diluted into GMML (200 mL) to be incubated at 37°C until OD_600_ = 0.4–0.6. IPTG was added to reach the final concentration of 0.5 mM, and non-canonical amino acid was added to reach the final concentration of 1 mM. Cultures were incubated at 18°C for 16 h with constant agitation for protein production. Protein was purified using Ni–NTA resin. The protein size was verified by SDS-PAGE.

### Enzyme kinetics measurements of PedH and variants

Purified PedH and variants activities were measured in 96-well microtiter plates with a dye-linked colorimetric assay referred to previous studies (Jahn et al. 2020; Wehrmann et al. 2020). Briefly, 200 µL of assay solution contained 100 mM Tris–HCl (pH 8), 1 mM phenazine ethosulfate (PES), 150 µM 2,6-dichlorophenol indophenol (DCPIP), 1.5 µM PrCl_3_, 1.5 µM PQQ, and 0.5 µM of enzyme. Before adding substrate, the enzyme and the assay solution were incubated at 30°C in the microplate reader until the background reaction disappeared. Enzyme activity was calculated by the change of OD_600_ within the first minute after the addition of substrate. The results were expressed as the mean ± standard deviation (*n* = 3). Kinetic parameters of PedH and its mutant were determined by fitting the enzyme activities at different substrate concentrations to the Michaelis–Menten equation. Kinetic values are expressed as average value with corresponding standard error.

### LC–MS of intact purified proteins

The specific molecular weight of the protein was obtained using matrix-assisted laser ionization time-of-flight mass spectrometry (MALDI-TOF–MS, Produced by Zhejiang Shuren University). LC–ESI–MS was performed using an Agilent 1290 Infinity II LC system connected to a 6545 Q-TOF mass spectrometer (Agilent, UK). Samples of 5 µL protein at 0.4 µg/µL were injected onto an Agilent PLRP-S (50 mm × 2.1 mm, 1000 Å, 5 µm) column, maintained at 30°C. Two mobile phases A (5% MeCN in aqueous 0.1% formic acid) and B (95% MeCN, 5% water, 0.1% formic acid) were used at 0.3 mL/min. The column was pre-equilibrated at 15% B for 1.9 min, before injection, held for 1 min further at 15% B, and then a gradient elution increased B to 90% over 16 min. After 2 min, B was decreased to 15% over 0.1 min. The QTOF mass spectrometer scanned m/z from 100 to 3100 Da. Positive electrospray ionization (ESI) was used with 4000 V capillary voltage, nozzle voltage at 500 V, fragmentor at 175 V, skimmer at 65 V and octopole RF peak at 750 V. Nitrogen was used as the nebulizer with nebulizer pressure at 45 psi and desolvation gas at a flow of 5 L/min. Spectra were acquired every second with an acquisition time of 1000 ms/spectrum. Lockspray was used during analysis to maintain mass accuracy. Data were processed in MassHunter Bioconfirm software (version B.10.00) and deconvolved using the maximum entropy deconvolution algorithm. Theoretical masses of wild-type proteins were calculated using Protparam (http://us.expasy.org/tools/protparam.html), and theoretical masses for unnatural amino acid containing proteins adjusted manually.

### Protein structure prediction

Alphafold2-multimer (https://github.com/deepmind/alphafold) was applied to predict the structure of *Mb*PylRS with high quality following its official protocol. The predicted structure was submitted to the UCLA - DOE LAB-SAVES v6.0 web server (https://saves.mbi.ucla.edu/) for quality evaluation. Virtual mutations were performed by Chimera software. Structures were visualized using Pymol (Schrodinger 2015).

### Molecular docking

The structure of *Mb*PylRS was superimposed with the *Mm*PylRS NTD-tRNA^Pyl^ complex (PDB: 5UD5) and the *Mm*PylRS CTD-ATP-Cyc complex (PDB: 2Q7G) to obtain the *Mb*PylRS-tRNA^Pyl^ protein acceptor. The predicted binding sites for the substrate ncAAs were recognized by the Autodock vina software. The docking was carried out according to the manufacturer’s protocol with default settings. Molecular docking of PedH with the substrate ethanol or methanol was similar to the method described above.

### Molecular dynamics simulation analysis of PedH variants

The X-ray crystal structure of PedH was taken from RCSB Crystal Database (PDB ID: 6ZCW) (Wehrmann et al. 2020). The initial structure of MD simulations contains a single-chain quinoprotein ethanol dehydrogenase, two ligands (PQQ and Praseodymium ion) and a substrate. The substrates were docked by Autodock Vina. MD simulations were performed using CHARMM36 protein force fields within GROMACS 2023 package (Huang and MacKerell 2013; Abraham et al. 2015). The force field parameters for PQQ were constructed by the CGenFF web server using the CHARMM general force field (Yu et al. 2012). The force constant of the harmonic biasing potential was 1000 kJ mol^−1^ nm^−2^. Three atoms (O3, O4, and N2) in the PQQ ligand were restrained by harmonic restraining potentials in the direction of lanthanide ions with an equilibrium distance of 0.25 nm and harmonic force constant of 9000 kJ mol^−1^ nm^−2^. The substrate butanol and methanol were restrained by harmonic restraining potentials in the same direction and used the same harmonic force constant with an equilibrium distance of 0.35 nm and 0.43 nm, respectively. The TIP3P water model was used to solvate the protein–ligand complex in a rectangular box that has a 10 Å distance to the largest protein boundary. Chloride ions were added to the solution to maintain the system’s neutrality.

The simulations are carried out for seven PedH systems with PQQ at 300 K, including wild-type /F412OBT PedH docked with (*S*)-2-butanol/(*R*)-2-butanol, and also wild-type/F412PLA/M439A PedH docked with methanol. At the start, the system was minimized for 10000 steps using the steepest descent algorithm. In the pre-equilibrium stage, the system was gradually heated to 300 K using 200 ps in the NVT ensemble and 200 ps in the NPT ensemble at 1 atm. Then, the NPT ensemble was further employed for 300 ns to equilibrate the system at 300 K and 1 atm, followed by a ten ns production run for collecting equilibrated configurations at each ten ps interval. The velocity-rescaling thermostat with a time constant equal to 0.1 ps was employed throughout the simulations to keep the temperature constant. To maintain the pressure, the Berendsen pressure coupling was used in the pre-equilibrium run, and the Parrinello–Rahman pressure coupling was utilized in the equilibrium and production run, with the pressure time constant and isothermal compressibility set to 2 ps and 4.5 $\times$ 10^–5^ bar^−1^, respectively. A time step of 2 fs for integration of the equations of motion was used throughout the simulation. A cutoff of 12 Å was used for non-bonded interactions. The particle mesh Ewald algorithm was used to calculate long-range electrostatic interactions. AmberTools was used to analyze the root-mean-square deviation (RMSD) of the protein backbone atoms relative to the initial structure. The root-mean-square fluctuations (RMSF) of backbone atoms were analyzed with the last 10 ns trajectories. The binding free energy was calculated using the MM/GBSA (molecular mechanics/generalized Born surface area) method.

### Supplementary Information

Additional file 1: **Table S1.** Kinetic parameters of PedH and its variant toward methanol, (*S*)-2-butanol and (*R*)-2-butanol. **Table S2.** Primers used for N-terminal engineering of *Mb*PylRS. **Table S3.** Primers for introducing amber codon to F412 site. **Fig. S1.** plDDT scores of five structures predicted by Alphafold2-multimer. **Fig. S2.** Dimeric structure of *Mb*PylRS predicted by Alphafold2-multimer. **Fig. S3.** Assessment of the rank_1 model without the variable linker using the SAVES v6.0 sever. **Fig. S4.** Sequence alignment of *Methanosarcina barkeri* PylRS and *Methanosarcina mazei* PylRS. **Fig. S5.** The secondary structure of tRNA^Pyl^ from *Methanosarcina barkeri* and *Methanosarcina mazei*. **Fig. S6.** Structures of the 43 novel ncAAs that tested by IPE variant. **Fig. S7.** SDS-PAGE analysis of sfGFP incorporated with the 16 novel ncAAs. **Fig. S8.** Deconvoluted ESI-MS spectrum of the purified full-length sfGFP proteins. **Fig. S9.** Superposition of the **23**/**24**-IPE complex over the *Mm*PylRS CTD-ATP-Cyc complex (PDB: 2Q7G). **Fig. S10.** SDS-PAGE analysis of PedH incorporated with different ncAAs. **Fig. S11.** Structural features of the ethanol-PedH binding sites and residue conformations of ncAAs after incorporation into the F412 site predicted by Chimera.** Fig. S12.** MD simulations to analyze the enantioselectivity difference of the mutant F412OBT with *O*-tert-Butyl-L-tyrosine (**8**) incorporated. **Fig. S13.** Interactions between substrates and protein residues analyzed in the MD simulations for wild type and mutant F412OBT.** Fig. S14.** Exploration of the effect of phenyllactic acid on enzyme catalysis. **Fig. S15.** RMSF values of 412 site calculated for the backbone atoms of wild type and F412PLA mutant.

## Data Availability

All data generated or analyzed during this study are included in this published article (and its Additional information files).

## References

[CR1] Abraham MJ, Murtola T, Schulz R, Páll S, Smith JC, Hess B, Lindahl E (2015). GROMACS: high performance molecular simulations through multi-level parallelism from laptops to supercomputers. SoftwareX.

[CR2] Brittain WDG, Lloyd CM, Cobb SL (2020). Synthesis of complex unnatural fluorine-containing amino acids. J Fluor Chem.

[CR3] Brown W, Liu J, Deiters A (2018). Genetic code expansion in animals. ACS Chem Biol.

[CR4] Bryson DI, Fan C, Guo LT, Miller C, Soll D, Liu DR (2017). Continuous directed evolution of aminoacyl-tRNA synthetases. Nat Chem Biol.

[CR5] Cobb SL, Murphy CD (2009). ^19^F NMR applications in chemical biology. J Fluorine Chem.

[CR6] Cui Y, Chen Y, Liu X, Dong S, Ye T, Qiao Y, Mitra R, Han J, Li C, Han X, Liu W, Chen Q, Wei W, Wang X, Du W, Tang S, Xiang H, Liu H, Liang Y, Houk KN, Wu B (2021). Computational redesign of a PETase for plastic biodegradation under ambient condition by the GRAPE strategy. ACS Catal.

[CR7] Englert M, Nakamura A, Wang YS, Eiler D, Soll D, Guo LT (2015). Probing the active site tryptophan of *Staphylococcus aureus* thioredoxin with an analog. Nucleic Acids Res.

[CR8] Fuhrer TJ, Houck M, Corley CA, Iacono ST (2019). Theoretical explanation of reaction site selectivity in the addition of a phenoxy group to perfluoropyridine. J Phys Chem A.

[CR9] Good NM, Vu HN, Suriano CJ, Subuyuj GA, Skovran E, Martinez-Gomez NC (2016). Pyrroloquinoline quinone ethanol dehydrogenase in *Methylobacterium extorquens* AM1 extends lanthanide-dependent metabolism to multicarbon substrates. J Bacteriol.

[CR10] Guo LT, Wang YS, Nakamura A, Eiler D, Kavran JM, Wong M, Kiessling LL, Steitz TA, O'Donoghue P, Soll D (2014). Polyspecific pyrrolysyl-tRNA synthetases from directed evolution. P Natl Acad Sci USA.

[CR11] Huang J, MacKerell AD (2013). CHARMM36 all-atom additive protein force field: validation based on comparison to NMR data. J Comput Chem.

[CR12] Jahn B, Pol A, Lumpe H, Barends TRM, Dietl A, Hogendoorn C, Op den Camp HJM, Daumann LJ (2018). Similar but not the same: first kinetic and structural analyses of a methanol dehydrogenase containing a europium ion in the active site. ChemBioChem.

[CR13] Jahn B, Jonasson NSW, Hu H, Singer H, Pol A, Good NM, den Camp H, Martinez-Gomez NC, Daumann LJ (2020). Understanding the chemistry of the artificial electron acceptors PES, PMS, DCPIP and Wurster’s blue in methanol dehydrogenase assays. J Biol Inorg Chem.

[CR14] Jiang HK, Lee MN, Tsou JC, Chang KW, Tseng HW, Chen KP, Li YK, Wang YS (2020). Linker and N-terminal domain engineering of pyrrolysyl-tRNA synthetase for substrate range shifting and activity enhancement. Front Bioeng Biotech.

[CR15] Kavran JM, Gundliapalli S, O'Donoghue P, Englert M, Soell D, Steitz TA (2007). Structure of pyrrolysyl-tRNA synthetase, an archaeal enzyme for genetic code innovation. P Natl Acad Sci USA.

[CR16] Keltjens JT, Pol A, Reimann J, Op den Camp HJ (2014). PQQ-dependent methanol dehydrogenases: rare-earth elements make a difference. Appl Microbiol Biotechnol.

[CR17] Koch NG, Goettig P, Rappsilber J, Budisa N (2021). Engineering Pyrrolysyl-tRNA synthetase for the incorporation of non-canonical amino acids with smaller side chains. Int J Mol Sci.

[CR18] Kurra Y, Odoi KA, Lee YJ, Yang Y, Lu T, Wheeler SE, Torres-Kolbus J, Deiters A, Liu WR (2014). Two rapid catalyst-free click reactions for in vivo protein labeling of genetically encoded strained alkene/alkyne functionalities. Bioconjug Chem.

[CR19] Li JC, Liu T, Wang Y, Mehta AP, Schultz PG (2018). Enhancing protein stability with genetically encoded noncanonical amino acids. J Am Chem Soc.

[CR20] Neumann H, Peak-Chew SY, Chin JW (2008). Genetically encoding *N*^ε^-acetyllysine in recombinant proteins. Nat Chem Biol.

[CR21] Owens AE, Grasso KT, Ziegler CA, Fasan R (2017). Two-tier screening platform for directed evolution of aminoacyl-tRNA synthetases with enhanced stop codon suppression efficiency. Chem Bio Chem.

[CR22] Schrodinger LLC (2015). The PyMOL molecular graphics system. Version.

[CR23] Shandell MA, Tan Z, Cornish VW (2021). Genetic code expansion: a brief history and perspective. Biochemistry-Us.

[CR24] Sharma V, Wang YS, Liu WR (2016). Probing the catalytic charge-relay system in alanine racemase with genetically encoded histidine mimetics. ACS Chem Biol.

[CR25] Sharma V, Zeng Y, Wang WW, Qiao Y, Kurra Y, Liu WR (2018). Evolving the N-terminal domain of pyrrolysyl-tRNA synthetase for improved incorporation of noncanonical amino acids. Chem Bio Chem.

[CR26] Spinck M, Piedrafita C, Robertson WE, Elliott TS, Cervettini D, de la Torre D, Chin JW (2023). Genetically programmed cell-based synthesis of non-natural peptide and depsipeptide macrocycles. Nat Chem.

[CR27] Suzuki T, Miller C, Guo LT, Ho JML, Bryson DI, Wang YS, Liu DR, Soll D (2017). Crystal structures reveal an elusive functional domain of pyrrolysyl-tRNA synthetase. Nat Chem Biol.

[CR28] Tharp JM, Wang YS, Lee YJ, Yang Y, Liu WR (2014). Genetic incorporation of seven *ortho*-substituted phenylalanine derivatives. ACS Chem Biol.

[CR29] Tharp JM, Ehnbom A, Liu WR (2018). tRNA^Pyl^: Structure, function, and applications. RNA Biol.

[CR30] Tuley A, Wang YS, Fang X, Kurra Y, Rezenom YH, Liu WR (2014). The genetic incorporation of thirteen novel non-canonical amino acids. Chem Commun (camb).

[CR31] Vatansever EC, Yang KS, Geng ZZ, Qiao Y, Li P, Xu S, Liu WR (2022). A designed, highly efficient pyrrolysyl-tRNA synthetase mutant binds *o*-chlorophenylalanine using two halogen bonds. J Mol Biol.

[CR32] Wan W, Tharp JM (1844). Liu WR (2014) pyrrolysyl-tRNA synthetase: an ordinary enzyme but an outstanding genetic code expansion tool. Bba-Proteins Proteom.

[CR33] Wang YS, Russell WK, Wang Z, Wan W, Dodd LE, Pai PJ, Russell DH, Liu WR (2011). The *de novo* engineering of pyrrolysyl-tRNA synthetase for genetic incorporation of L-phenylalanine and its derivatives. Mol Biosyst.

[CR34] Wang YS, Fang X, Wallace AL, Wu B, Liu WR (2012). A rationally designed pyrrolysyl-tRNA synthetase mutant with a broad substrate spectrum. J Am Chem Soc.

[CR35] Wehrmann M, Billard P, Martin-Meriadec A, Zegeye A, Klebensberger J (2017). Functional role of lanthanides in enzymatic activity and transcriptional regulation of pyrroloquinoline quinone-dependent alcohol dehydrogenases in *Pseudomonas putida* KT2440. Mbio.

[CR36] Wehrmann M, Elsayed EM, Köbbing S, Bendz L, Lepak A, Schwabe J, Wierckx N, Bange G, Klebensberger J (2020). Engineered PQQ-dependent alcohol dehydrogenase for the oxidation of 5-(Hydroxymethyl)furoic acid. ACS Catal.

[CR37] Wilkinson HC, Dalby PA (2021). Fine-tuning the activity and stability of an evolved enzyme active-site through noncanonical amino-acids. Febs J.

[CR38] Williams TL, Iskandar DJ, Nodling AR, Tan Y, Luk LYP, Tsai YH (2021). Transferability of N-terminal mutations of pyrrolysyl-tRNA synthetase in one species to that in another species on unnatural amino acid incorporation efficiency. Amino Acids.

[CR39] Young DD, Schultz PG (2018). Playing with the Molecules of Life. ACS Chem Biol.

[CR40] Young DD, Young TS, Jahnz M, Ahmad I, Spraggon G, Schultz PG (2011). An evolved aminoacyl-tRNA Synthetase with atypical polysubstrate specificity. Biochemistry-Us.

[CR41] Yu W, He X, Vanommeslaeghe K, MacKerell AD (2012). Extension of the CHARMM general force field to sulfonyl-containing compounds and its utility in biomolecular simulations. J Comput Chem.

[CR42] Yu H, Ma S, Li Y, Dalby PA (2022). Hot spots-making directed evolution easier. Biotechnol Adv.

