# Supplementary material for: An evolved pyrrolysyl-tRNA synthetase with polysubstrate specificity expands the toolbox for engineering enzymes with incorporation of noncanonical amino acids
Source: Bioresour Bioprocess. 2023 Dec 11;10(1):92. doi: 10.1186/s40643-023-00712-w (PMC10991234; doi:10.1186/s40643-023-00712-w)
Supplement: Supplementary file 1 — Additional file 1: Table S1. Kinetic parameters of PedH and its variant toward methanol, (S)-2-butanol and (R)-2-butanol. Table S2. Primers used for N-terminal engineering of MbPylRS. Table S3. Primers for introducing amber codon to F412 site. Fig. S1. plDDT scores of five structures predicted by Alphafold2-multimer. Fig. S2. Dimeric structure of MbPylRS predicted by Alphafold2-multimer. Fig. S3. Assessment of the rank_1 model without the variable linker using the SAVES v6.0 sever. Fig. S4. Sequence alignment of Methanosarcina barkeri PylRS and Methanosarcina mazei PylRS. Fig. S5. The secondary structure of tRNAPyl from Methanosarcina barkeri and Methanosarcina mazei. Fig. S6. Structures of the 43 novel ncAAs that tested by IPE variant. Fig. S7. SDS-PAGE analysis of sfGFP incorporated with the 16 novel ncAAs. Fig. S8. Deconvoluted ESI-MS spectrum of the purified full-length sfGFP proteins. Fig. S9. Superposition of the 23/24-IPE complex over the MmPylRS CTD-ATP-Cyc complex (PDB: 2Q7G). Fig. S10. SDS-PAGE analysis of PedH incorporated with different ncAAs. Fig. S11. Structural features of the ethanol-PedH binding sites and residue conformations of ncAAs after incorporation into the F412 site predicted by Chimera. Fig. S12. MD simulations to analyze the enantioselectivity difference of the mutant F412OBT with O-tert-Butyl-L-tyrosine (8) incorporated. Fig. S13. Interactions between substrates and protein residues analyzed in the MD simulations for wild type and mutant F412OBT. Fig. S14. Exploration of the effect of phenyllactic acid on enzyme catalysis. Fig. S15. RMSF values of 412 site calculated for the backbone atoms of wild type and F412PLA mutant. [file 40643_2023_712_MOESM1_ESM.docx]

**Additional Information**

An evolved pyrrolysyl-tRNA synthetase with polysubstrate specificity expands the toolbox for engineering enzymes with incorporation of noncanonical amino acids

*Ke Liu^a^, Ling Jiang^a,b^, Shuang Ma^a^, Zhongdi Song^c^*, Lun Wang^a,b^, Qunfeng Zhang^a^, Renhao Xu^d^, Lirong Yang^a,b^, Jianping Wu^a,b^, Haoran Yu^a, b^**

^a^ Institute of Bioengineering, College of Chemical and Biological Engineering, Zhejiang University, Hangzhou, Zhejiang 310027, China.

^b^ ZJU-Hangzhou Global Scientific and Technological Innovation Centre, Hangzhou, Zhejiang, 311200, China.

^c^ Key Laboratory of Pollution Exposure and Health Intervention of Zhejiang Province, Interdisciplinary Research Academy, Zhejiang Shuren University, Hangzhou, Zhejiang 310015, China.

^d^ Hangzhou 14th Middle School, Hangzhou, Zhejiang 310006, China.

*Correspondence and requests for materials should be addressed to Haoran Yu (Email: [yuhaoran@zju.edu.cn](mailto:yuhaoran@zju.edu.cn)), Zhongdi Song (Email: zhongdisong@foxmail.com)

**Contents**

[**Additional Tables 3**](#_Toc135338594)

[Table S1 Kinetic parameters of PedH and its variant towards methanol, (*S*)-2-butanol and (*R*)-2-butanol 3](#_Toc135338595)

[Table S2 Primers used for N-terminal engineering of *Mb*PylRS 4](#_Toc135338596)

[Table S3 Primers for introducing amber codon to F412 site 5](#_Toc135338597)

[**Additional Figures 6**](#_Toc135338598)

[Fig. S1 plDDT scores of five structures predicted by Alphafold2-multimer. 6](#_Toc135338599)

[Fig. S2 Dimeric structure of *Mb*PylRS predicted by Alphafold2-multimer. 7](#_Toc135338600)

[Fig. S3 Assessment of the rank_1 model without the variable linker using the SAVES v6.0 sever. 7](#_Toc135338601)

[Fig. S4 Sequence alignment of *Methanosarcina barkeri* PylRS and *Methanosarcina mazei* PylRS. 8](#_Toc135338602)

[Fig. S5 The secondary structure of tRNA^Pyl^ from *Methanosarcina barkeri* and *Methanosarcina mazei*. 9](#_Toc135338603)

[Fig. S6 Structures of the 43 novel ncAAs that tested by IPE variant. 10](#_Toc135338604)

[Fig. S7 SDS-PAGE analysis of sfGFP incorporated with the 16 novel ncAAs. 11](#_Toc135338605)

[Fig. S8 Deconvoluted ESI-MS spectrum of the purified full-length sfGFP proteins. 12](#_Toc135338606)

[Fig. S9 Superposition of the **23**/**24**-IPE complex over the *Mm*PylRS CTD-ATP-Cyc complex (PDB: 2Q7G). 12](#_Toc135338607)

[Fig. S10 SDS-PAGE analysis of PedH incorporated with different ncAAs. 13](#_Toc135338608)

[Fig. S11 Structural features of the ethanol-PedH binding sites and residue conformations of ncAAs after incorporation into the F412 site predicted by Chimera. 13](#_Toc135338609)

[Fig. S12 MD simulations to analyze the enantioselectivity difference of the mutant F412OBT with *O*-tert-Butyl-L-tyrosine (**8**) incorporated.. 10](#_Toc135338604)

[Fig. S13 Interactions between substrates and protein residues analysed in the MD simulations for wild type and mutant F412OBT. 15](#_Toc135338611)

[Fig. S14 Exploration of the effect of phenyllactic acid on enzyme catalysis. 10](#_Toc135338604)

[Fig. S15 RMSF values of 412 site calculated for the backbone atoms of wild type and F412PLA mutant. 17](#_Toc135338612)

[**Supplementary texts 18**](#_Toc135338598)

[PedH mutants incorporated with ncAAs having *meta*-substituents. 18](#_Toc135338599)

[PedH mutants incorporated with ncAAs having *para*-substituents. 19](#_Toc135338600)

[PedH mutant incorporated with hydroxy acid. 20](#_Toc135338601)

[**References 21**](#_Toc135338598)

# Additional Tables

## Table S1 Kinetic parameters of PedH and its variant towards methanol, (*S*)-2-butanol and (*R*)-2-butanol

|  | Substrates | Enzymes | | |
| --- | --- | --- | --- | --- |
|  |  | PedH | F412PLA | F412OBT |
| *K*_m_ (mM) | methanol | 1.10±0.18 | 0.70±0.18 | - |
|  | (*S*)-2-butanol | 1.07 ± 0.18 | - | 0.39± 0.05- |
|  | (*R*)-2-butanol | 0.94± 0.13 | - | 0.22 ± 0.04 |
| *k*_cat_ (s^-1^) | methanol | 0.15±0.01 | 0.17±0.01 | - |
|  | (*S*)-2-butanol | 0.43± 0.01 | - | 0.31 ± 0.02 |
|  | (*R*)-2-butanol | 0.42 ± 0.01 | - | 0.17 ± 0.01 |
| *k*_cat_ / *K*_m_ (M^-1^ s^-1^) | methanol | 136.36±13.22 | 242.86±48.16 | - |
|  | (*S*)-2-butanol | 401.87 ± 58.26 | - | 794.87 ± 50.62 |
|  | (*R*)-2-butanol | 446.81±51.15 | - | 772.73 ± 95.04 |

## Table S2 Primers used for N-terminal engineering of *Mb*PylRS

| Mb NACA-F | CATGGTTGCTTTTGCTCAAATGGGCAGCGGCTGC |
| --- | --- |
| Mb NACA-R | TTGAGCAAAAGCAACCATGGTGAATTCTTCCAGG |
| Mm NACA-F | GCTGGCTTTTGCTCAAATGGGTAGCGGTTGTACACG |
| Mm NACA-R | TTTGAGCAAAAGCCAGCATGGTAAATTCTTCCAGATG |
| V8E-F | CGCTGGATGAACTGATTAGCGCGACCGGCCTG |
| V8E-R | AATCAGTTCATCCAGCGGTTTTTTATCCATCA |
| T13I-F | ATTAGCGCGATTGGCCTGTGGATGAGCCGTAC |
| T13I-R | AGGCCAATCGCGCTAATCAGCACATCCAGCGG |
| I36V-F | GCCGCAGCAAAGTTTATATTGAAATGGCGTGCGGC |
| I36V-R | ATAAACTTTGCTGCGGCTCACTTCATGATGTT |
| H45L-F | GATCTGCTGGTGGTGAACAACAGCCGTAGCTG |
| H45L-R | TTCACCACCAGCAGATCGCCGCACGCCATTTC |
| S121R-F | GGAAAATCGTGTGAGCGCGAAAGCGAGCACCA |
| S121R-R | CGCTCACACGATTTTCCAGCGGTTTCGGCGCA |
| D76G-F | TGTGAGCGGCGAAGATATCAACAACTTTCTGACCCG |
| D76G-R | TATCTTCGCCGCTCACACGGCAACGTTTGCAG |
| V31I-F | CATGAAATTAGCCGCAGCAAAATCTATATTGA |
| V31I-R | CTGCGGCTAATTTCATGATGTTTGATTTTATGCAGG |
| T56P-F | GTCCGGCGCGTGCGTTTCGTCAT |
| T56P-R | AAACGCACGCGCCGGACGGCAGCTACGGCTGTTG |
| Y62H-F | GTGCGTTTCGTCATCATAAATACCGCAAAACCTGCA |
| Y62H-R | ATGATGACGAAACGCACGCGCCGGACGGCAGC |
| H62Y-2F | GTGCGTTTCGTCATTATAAATACCGCAAAACCTGCAAA |
| H62Y-2R | ATAATGACGAAACGCACGCGCCGGACGGCAGC |
| A100E-F | TGGTGAGCGAACCGAAAGTGAAAAAAGCGATG |
| A100E-R | TTTCGGTTCGCTCACCACACGCACTTTCA |

## Table S3 Primers for introducing amber codon to F412 site

| F412TAG-F | GCAtagCTGGGTGCAAAAAATTGGATGCCGAT |
| --- | --- |
| F412TAG-R | GCAtagCTGGGTGCAAAAAATTGGATGCCGAT |

# Additional Figures


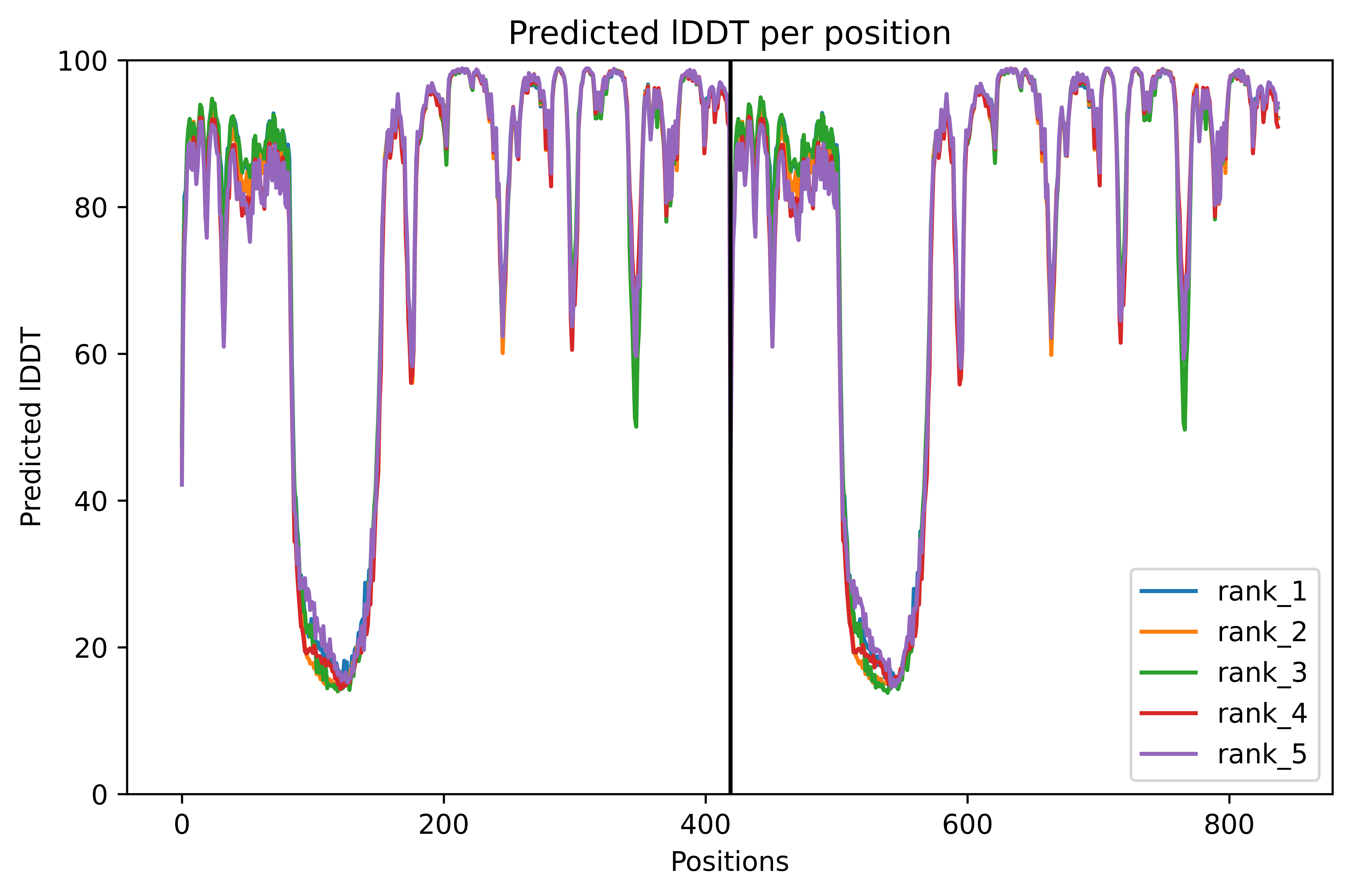


## Fig. S1 plDDT scores of five structures predicted by Alphafold2-multimer.


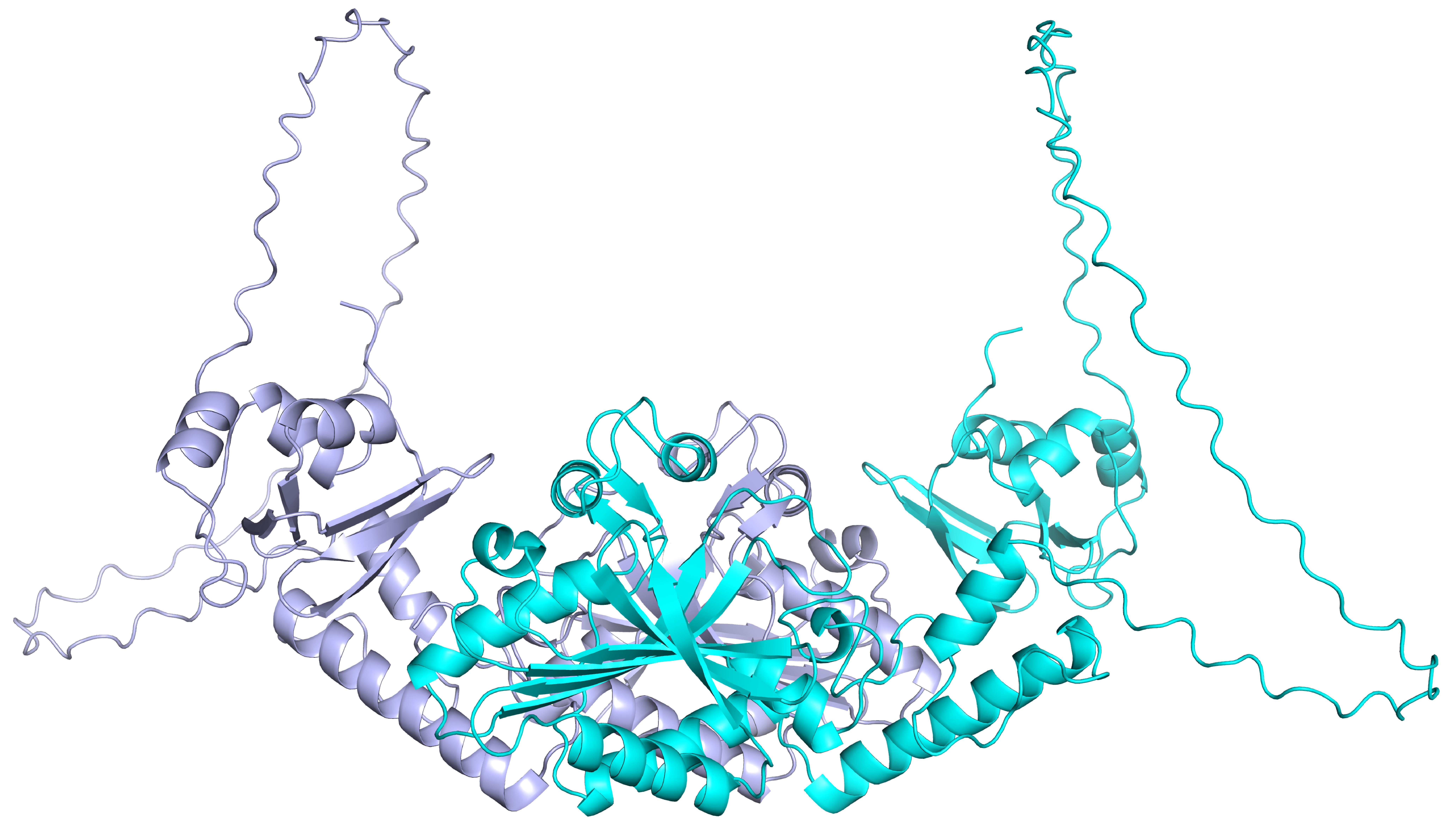


## Fig. S2 Dimeric structure of *Mb*PylRS predicted by Alphafold2-multimer.


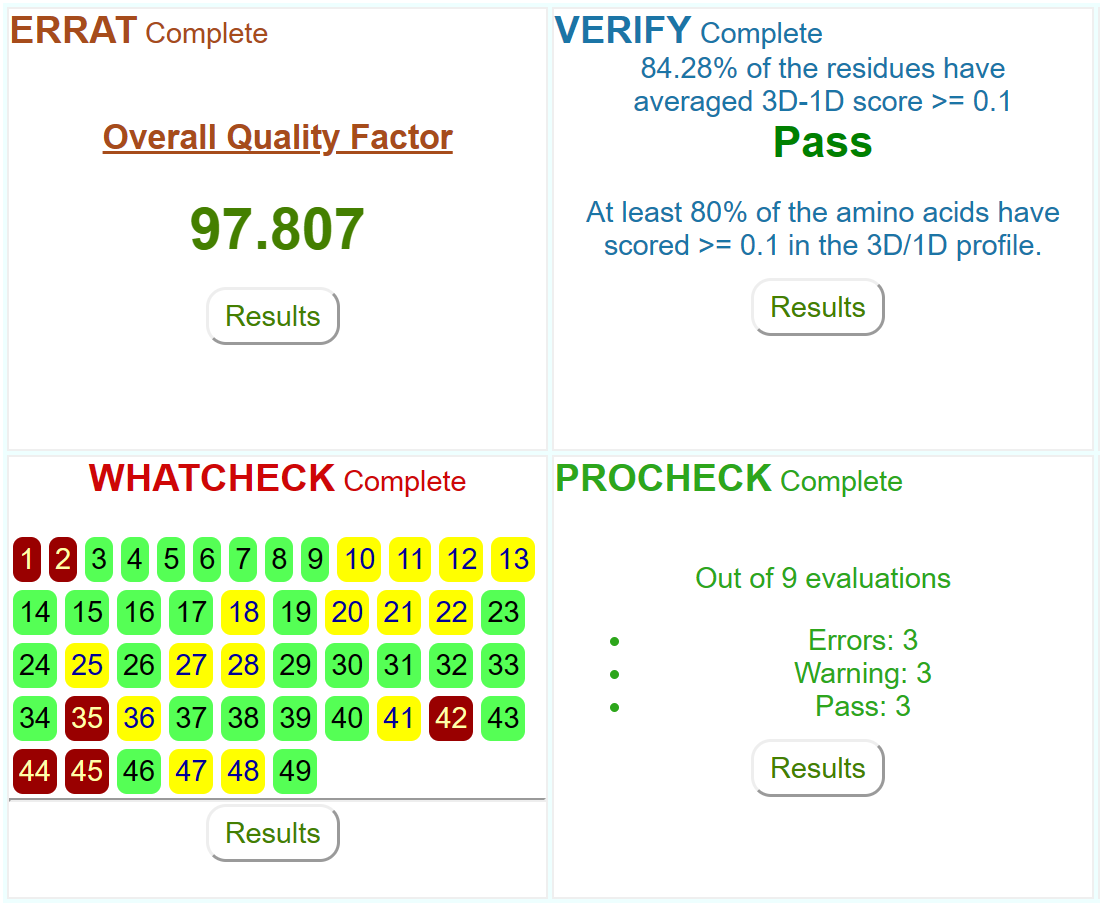


## Fig. S3 Assessment of the rank_1 model without the variable linker using the SAVES v6.0 sever.


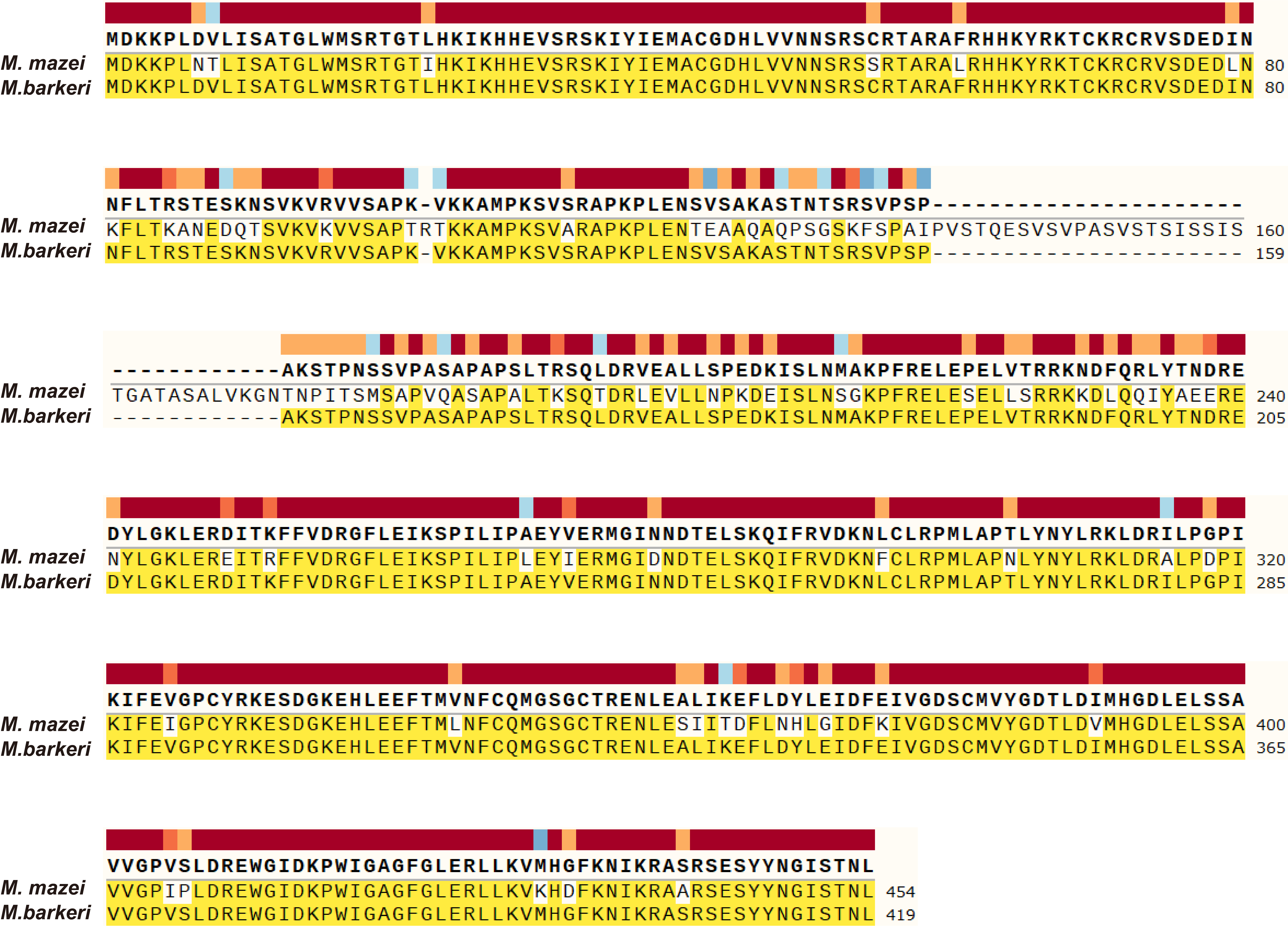


## Fig. S4 Sequence alignment of *Methanosarcina barkeri* PylRS and *Methanosarcina mazei* PylRS.


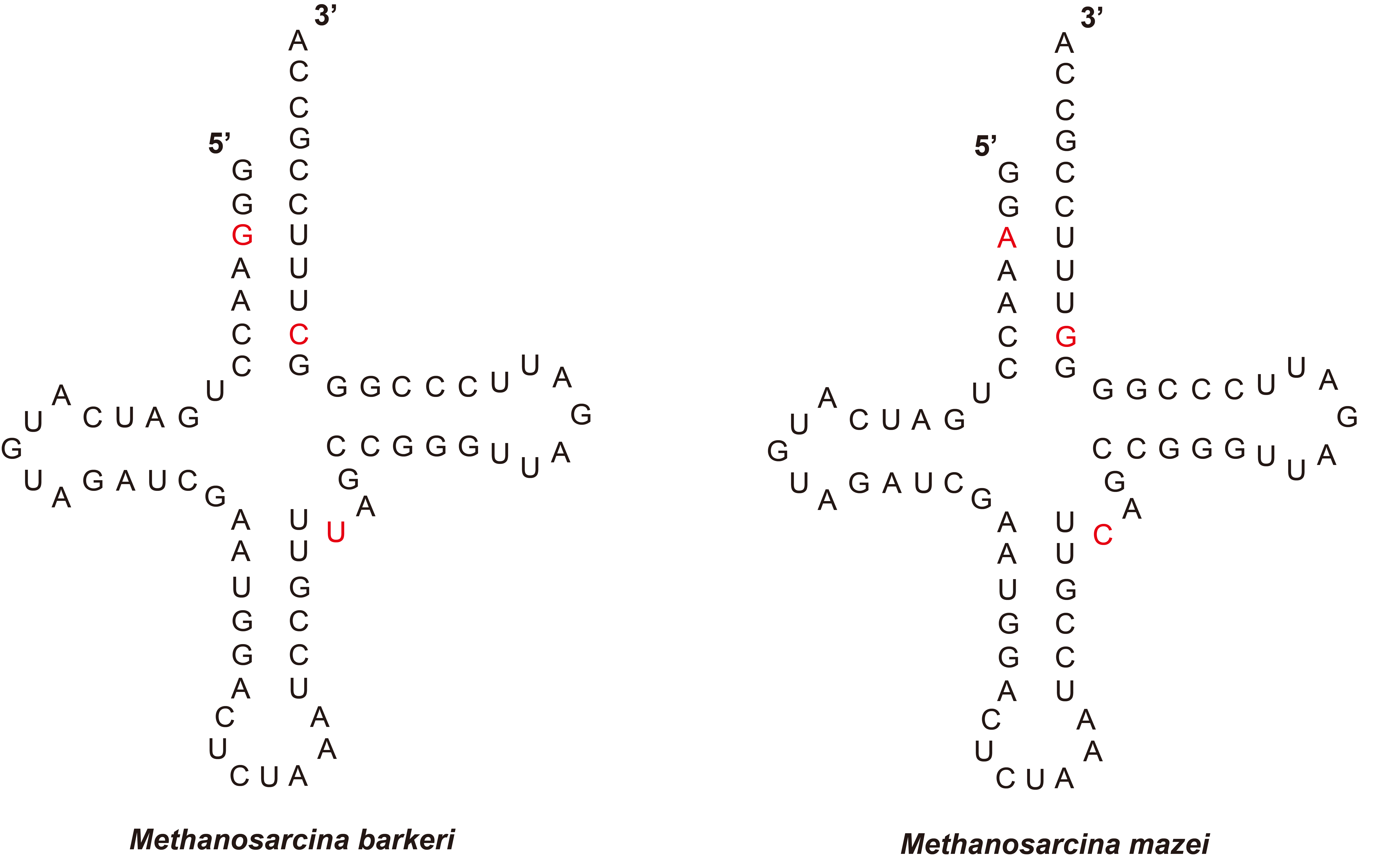


Fig. S5 The secondary structure of tRNA^Pyl^ from *Methanosarcina barkeri* and *Methanosarcina mazei*. The different bases are marked in red.

Fig. S6 Structures of the 43 novel ncAAs that tested by IPE variant. **13**, L-2,5-dichloroPhe; **14**, L-2,4-difluoroPhe; **15**, L-2,3-difluoroPhe; **16**, L-2,5-difluoroPhe; **17**, L-2,4,5-trifluoroPhe; **18**, L-2,3,6-trifluoroPhe; **19**, L-2,3-dichloroPhe; **20**, 5-bromo-2-chloro-L-Phe; **21**, L-2-(5-bromothienyl)alanine; **22**, L-3-benzothienylalanine; **23**, 3-(3-thienyl)-L-alanine; **24**, 3-(2-thienyl)-L-alanine; **25**, Phenyllactic acid; **26**, 2-amino-2-phenylpropionic acid; **27**, L-homoPhe; **28**, 4-nitro-L-Phe; **29**, 4-fluoro-L-Phe; **30**, L-2,3,4,5,6-pentafluoroPhe; **31**, L-3,4-difluoroPhe; **32**, 3-fluoro-L-Phe; **33**, 3-(1-naphthyl)-L-alanine; **34**, 2-amino-3-(3,4,5-trifluorophenyl)propanoic acid hcl; **35**, L-3,5-dichloroPhe; **36**, 4-bromo-2-chloro-L-Phe; **37**, 2-chloro-L-Phe; **38**, 3-bromo-2-methyl-L-Phe; **39**, 3-bromo-L-tyrosine; **40**, 3-iodo-L-tyrosine; **41**, 3-(methylseleno)-L-alanine; **42**, S-methyl-L-cysteine; **43**, 2-methyl-L-Phe; **44**, 4-methyl-L-leucine; **45**, 1-methyl-L-tryptophan; **46**, Ne-Boc-L-lysine; **47**, 3-(3-benzothienyl)-D-alanine; **48**, (S)-2-amino-3-cyclobutylpropanoic acid; **49**, 3-cyclopentane-L-alanine; **50**, (S)-2-amino-4-pentenoic acid; **51**, 3,5-dimethylcyclopentane-1,2-dione; **52**, 3-cyclopropyl-L-alanine; **53**, S-benzyl-L-cysteine; **54**, 4-amino-L-Phe; **55**, 4-cyano-L-Phe;


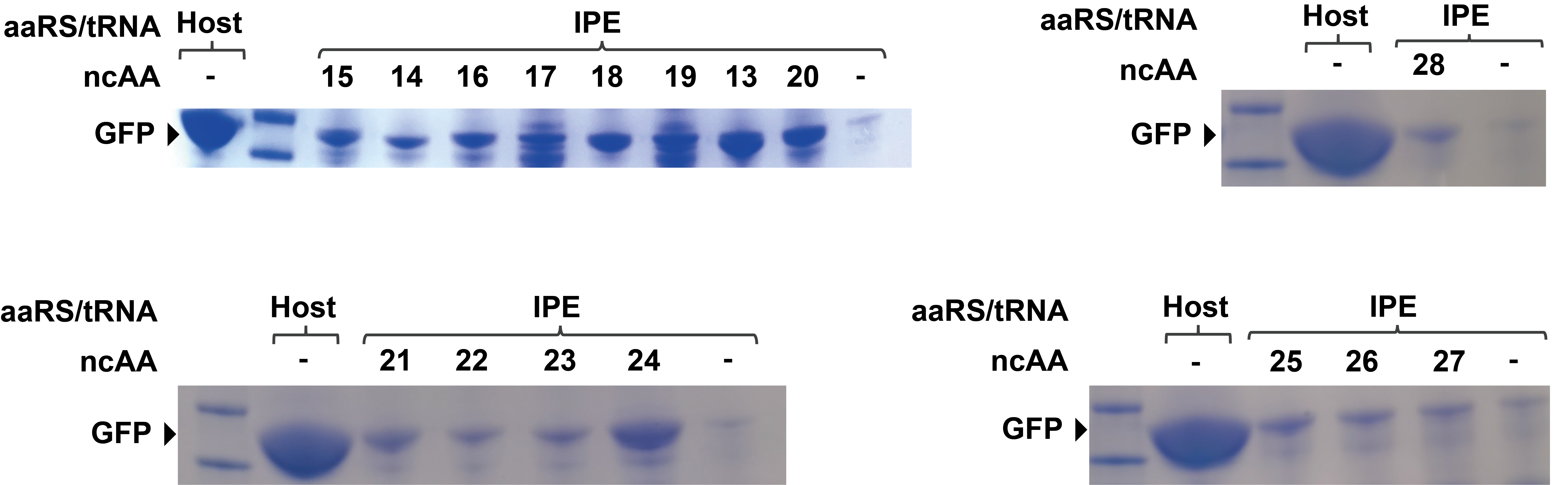


## Fig. S7 SDS-PAGE analysis of sfGFP incorporated with the 16 novel ncAAs.


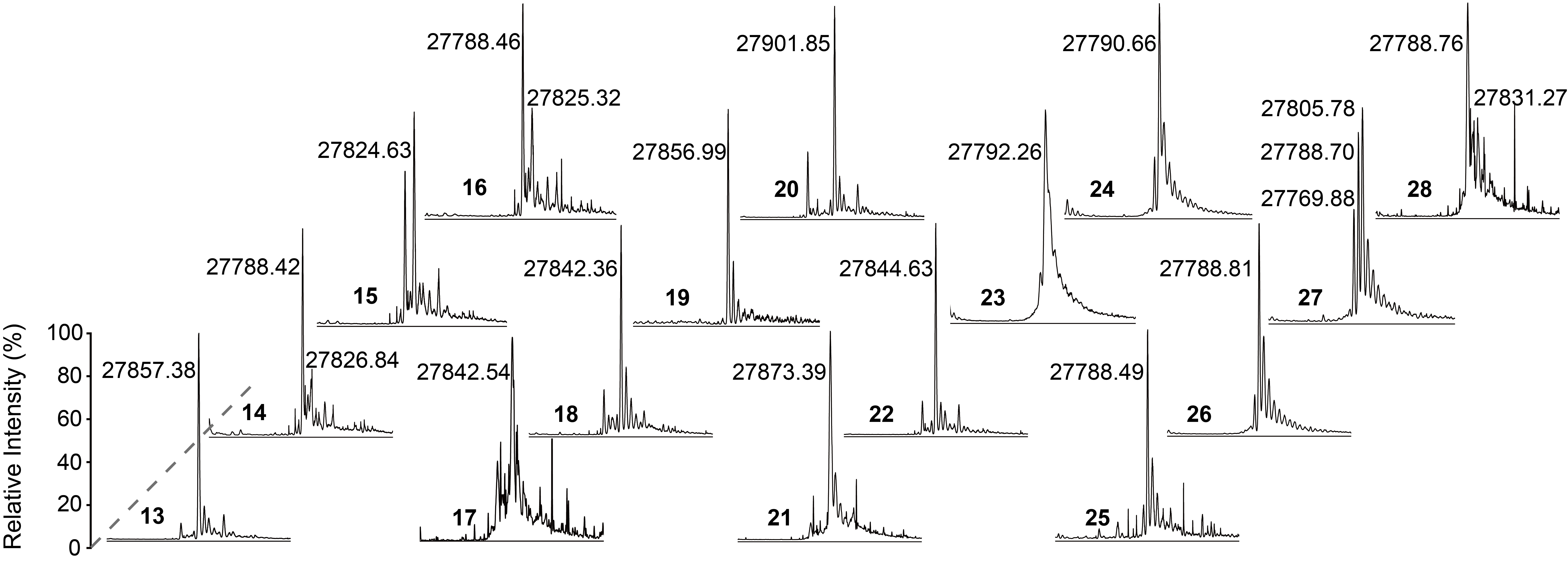


## Fig. S8 Deconvoluted ESI-MS spectrum of the purified full-length sfGFP proteins.


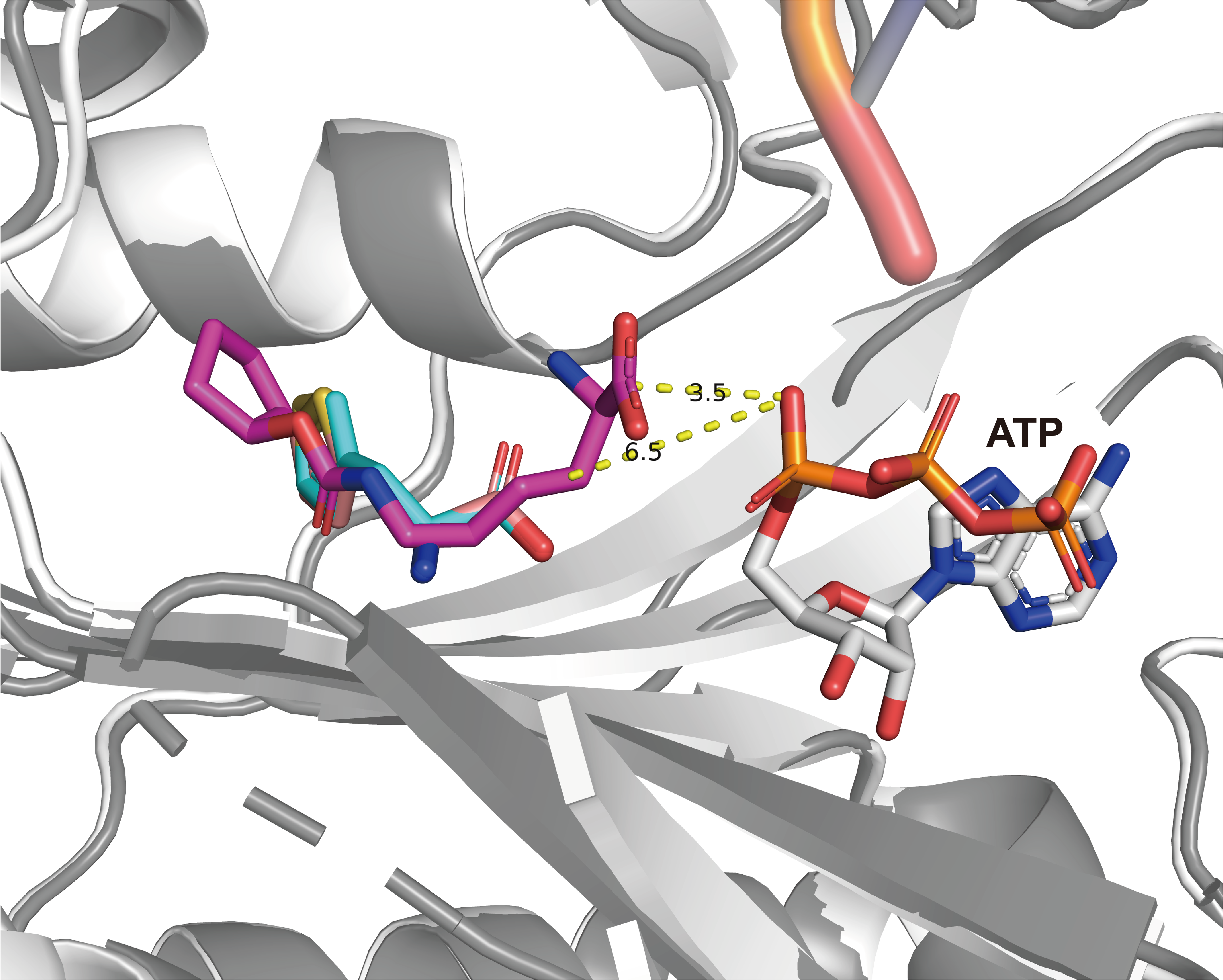


Fig. S9 Superposition of the 23/24-IPE complex over the *Mm*PylRS CTD-ATP-Cyc complex (PDB: 2Q7G). IPE is colored in white and *Mm*PylRS CTD is colored in gray. 3-(3-thienyl)-L-alanine (**23**) is represented as cyans sticks, 3-(2-thienyl)-L-alanine (**24**) is represented as salmon sticks, and Cyc is represented as magentas sticks.


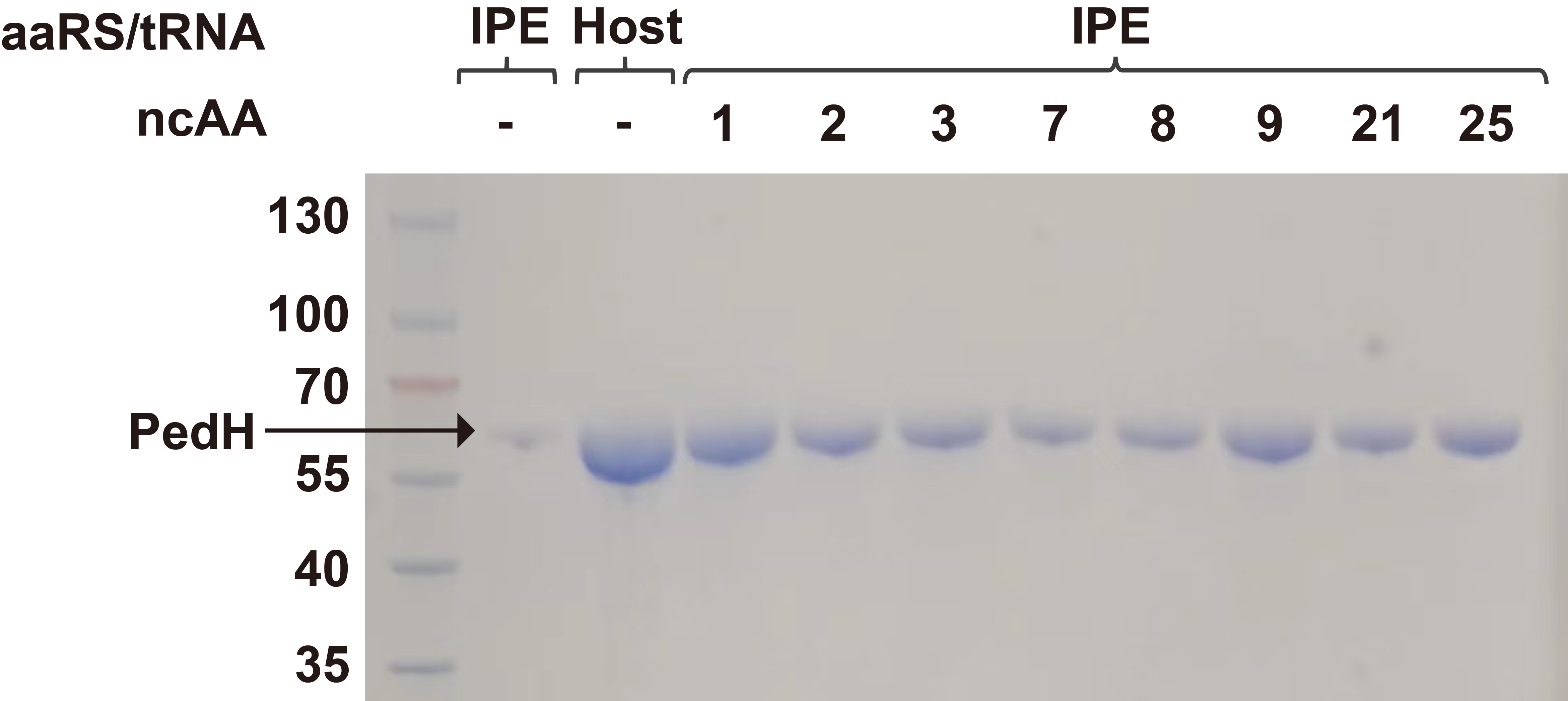


## Fig. S10 SDS-PAGE analysis of PedH incorporated with different ncAAs.


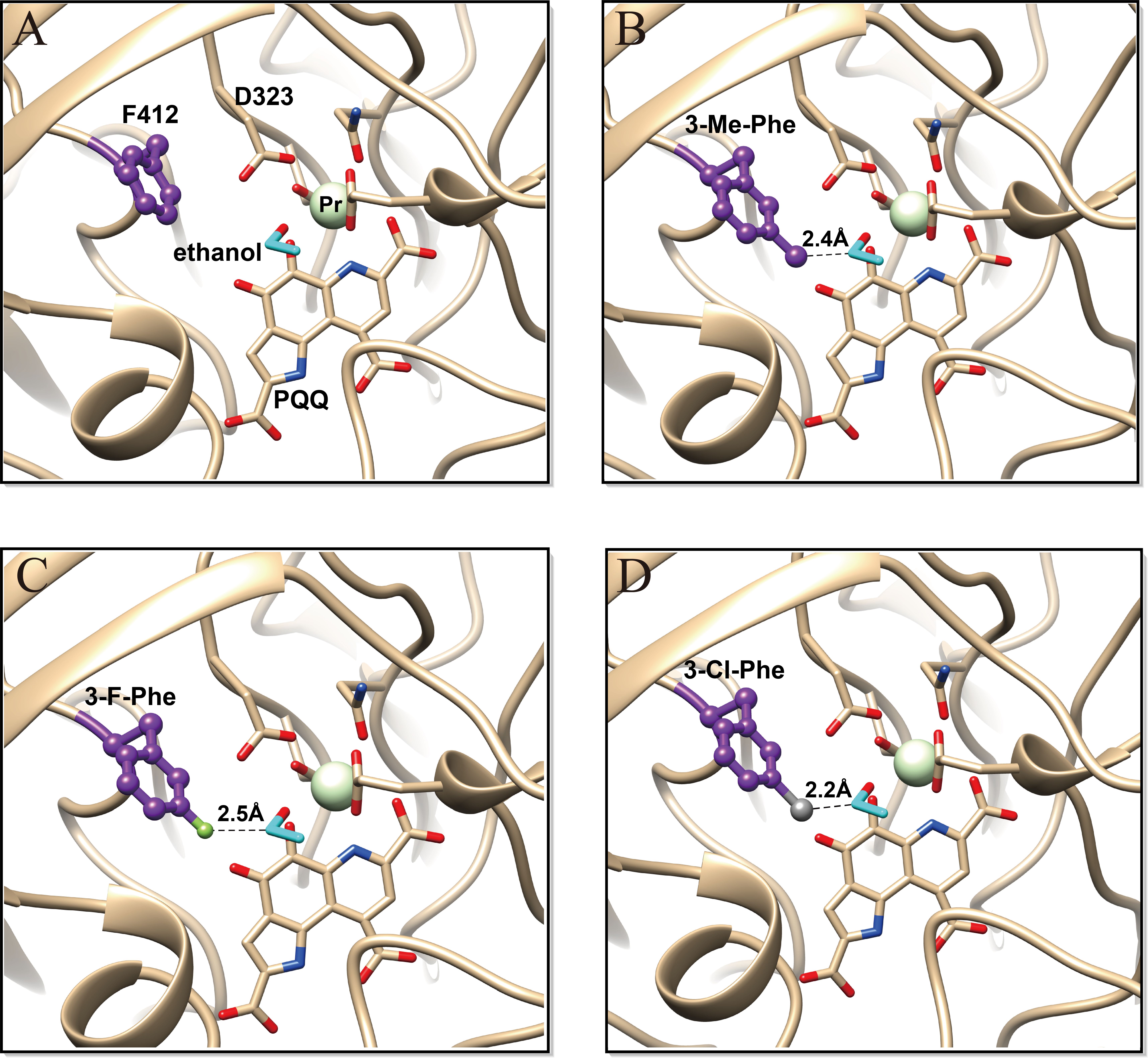


Fig. S11 Structural features of the ethanol-PedH binding sites and residue conformations of ncAAs after incorporation into the F412 site predicted by Chimera. (A) The ethanol-PedH binding sites predicted by Autodock vina. (B) The residue conformation after incorporating 3-Me-Phe (**3**) into the F412 site. (C) The residue conformation after incorporating 3-F-Phe into the F412 site. (D) The residue conformation after incorporating 3-Cl-Phe into the F412 site.


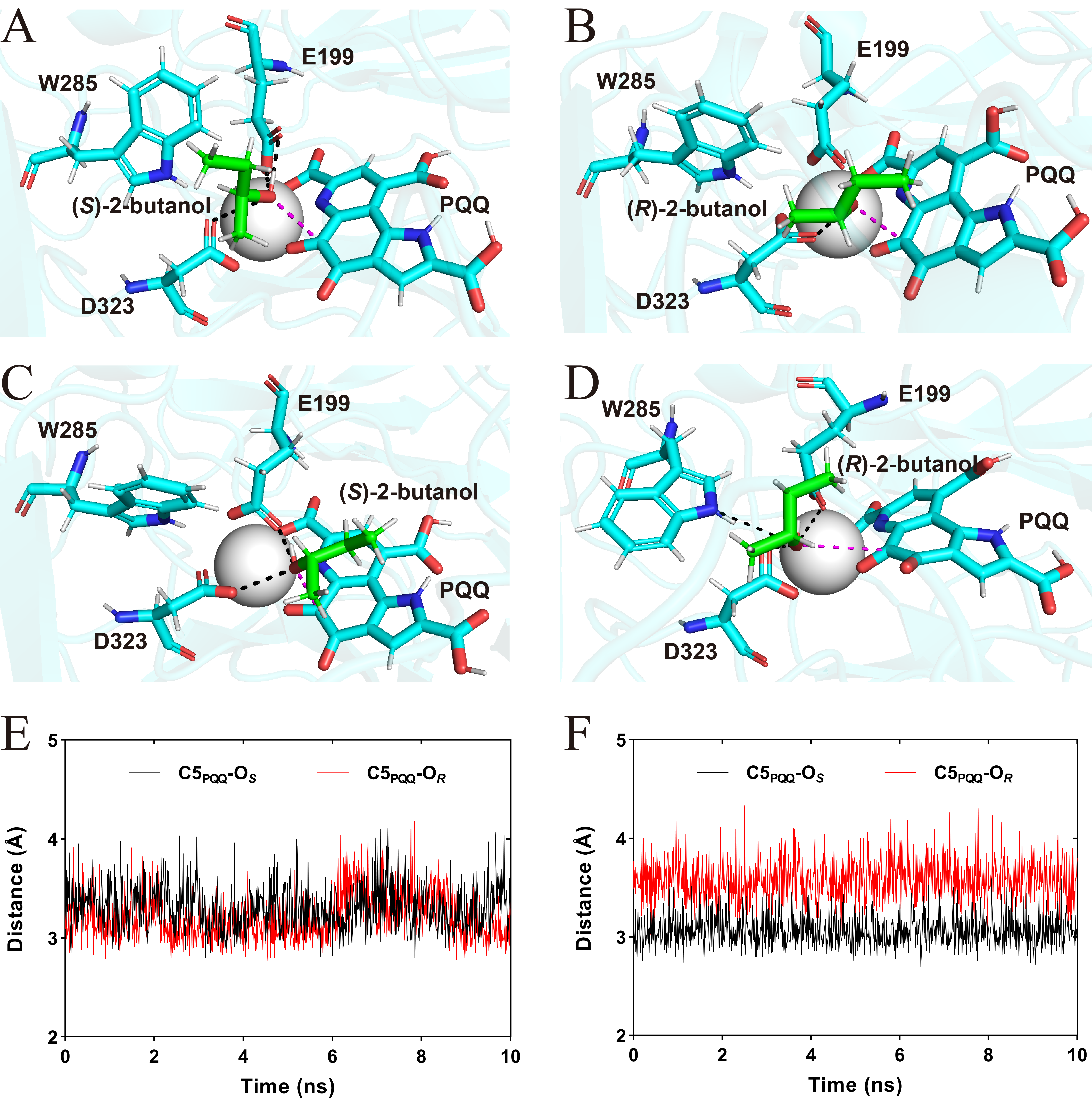


Fig. S12 MD simulations to analyze the enantioselectivity difference of the mutant F412OBT with *O*-tert-Butyl-L-tyrosine (**8**) incorporated. (A) Interactions between (*S*)-2-butanol and binding residues of the wild type. Black dotted lines indicate hydrogen bonds and the magenta dotted line connects the oxygen atom of substrate to C5 of cofactor PQQ. (B) Interactions between (*R*)-2-butanol and binding residues of the wild type. (C) Interactions between (*S*)-2-butanol and binding residues of F412OBT. (D) Interactions between (*R*)-2-butanol and binding residues of F412OBT. (E) Distance between oxygen atom of substrate and C5 of PQQ for wild type. (F) Distance between oxygen atom of substrate and C5 of PQQ for F412OBT.


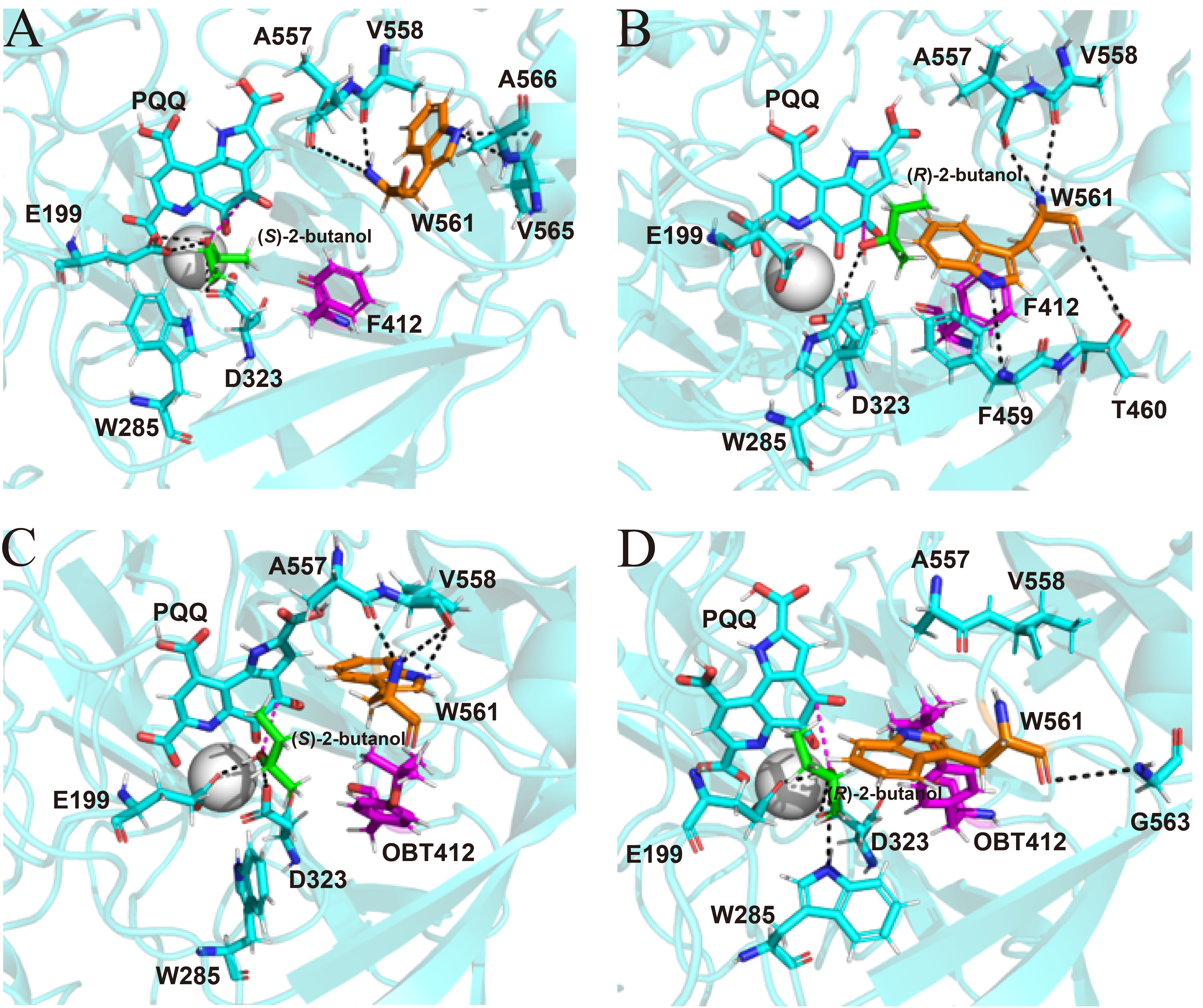


Fig. S13 Interactions between substrates and protein residues analysed in the MD simulations for wild type and mutant F412OBT. (A) WT-(*S*)-2-butanol. (B) WT-(*R*)-2-butanol. (C) F412OBT-(*S*)-2-butanol. (D) F412OBT-(*R*)-2-butanol.


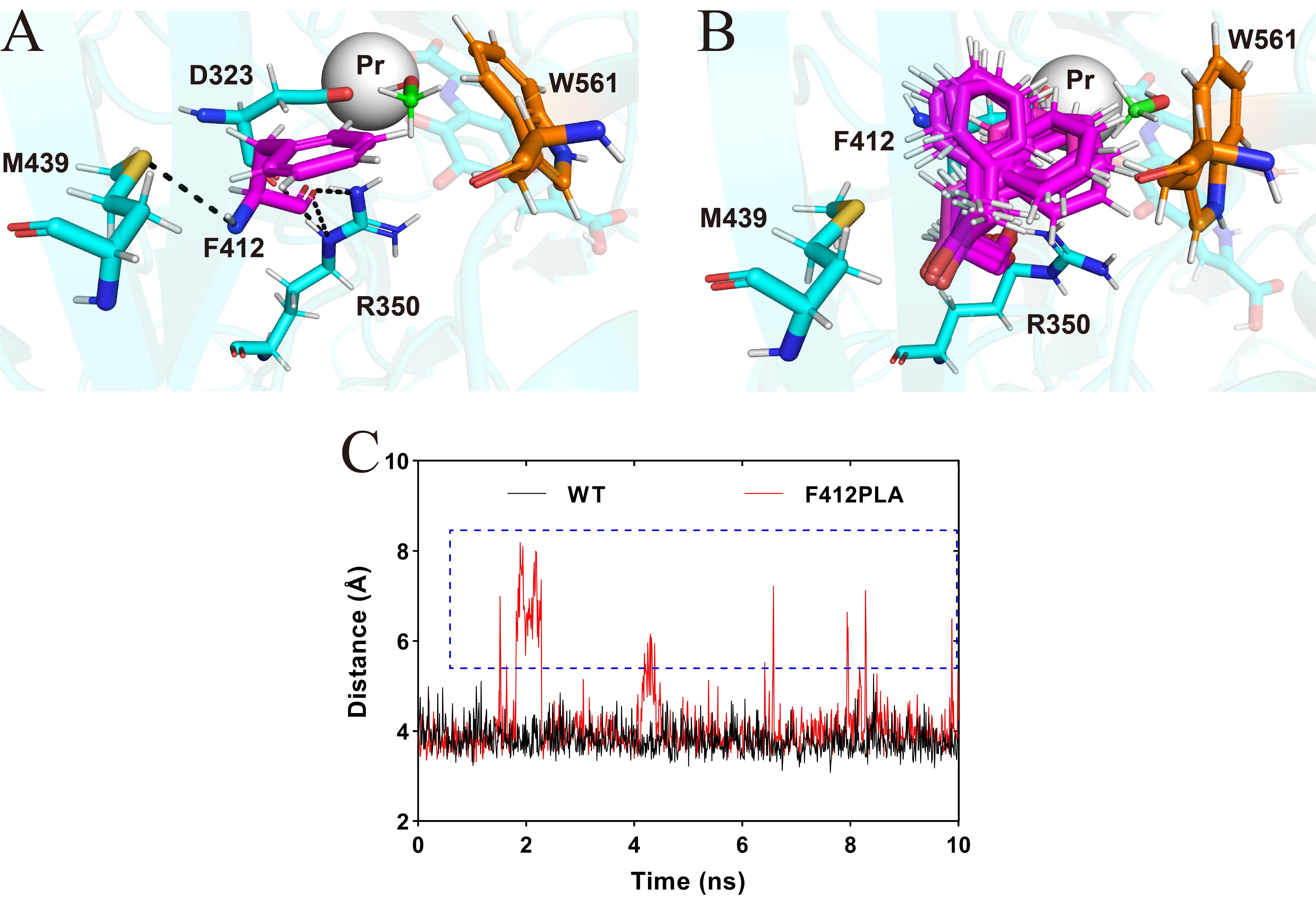


Fig. S14 Exploration of the effect of phenyllactic acid on enzyme catalysis. (A) Hydrogen bonds between F412 and surrounding residues in F412PLA-methanol complex. (B) Different conformations of PLA412 observed in the MD simulations of the F412PLA-methanol complex. (C) The distance between 412 site C_α_ and 561 site C_α_ measured in the MD simulations of the F412PLA-methanol complex.


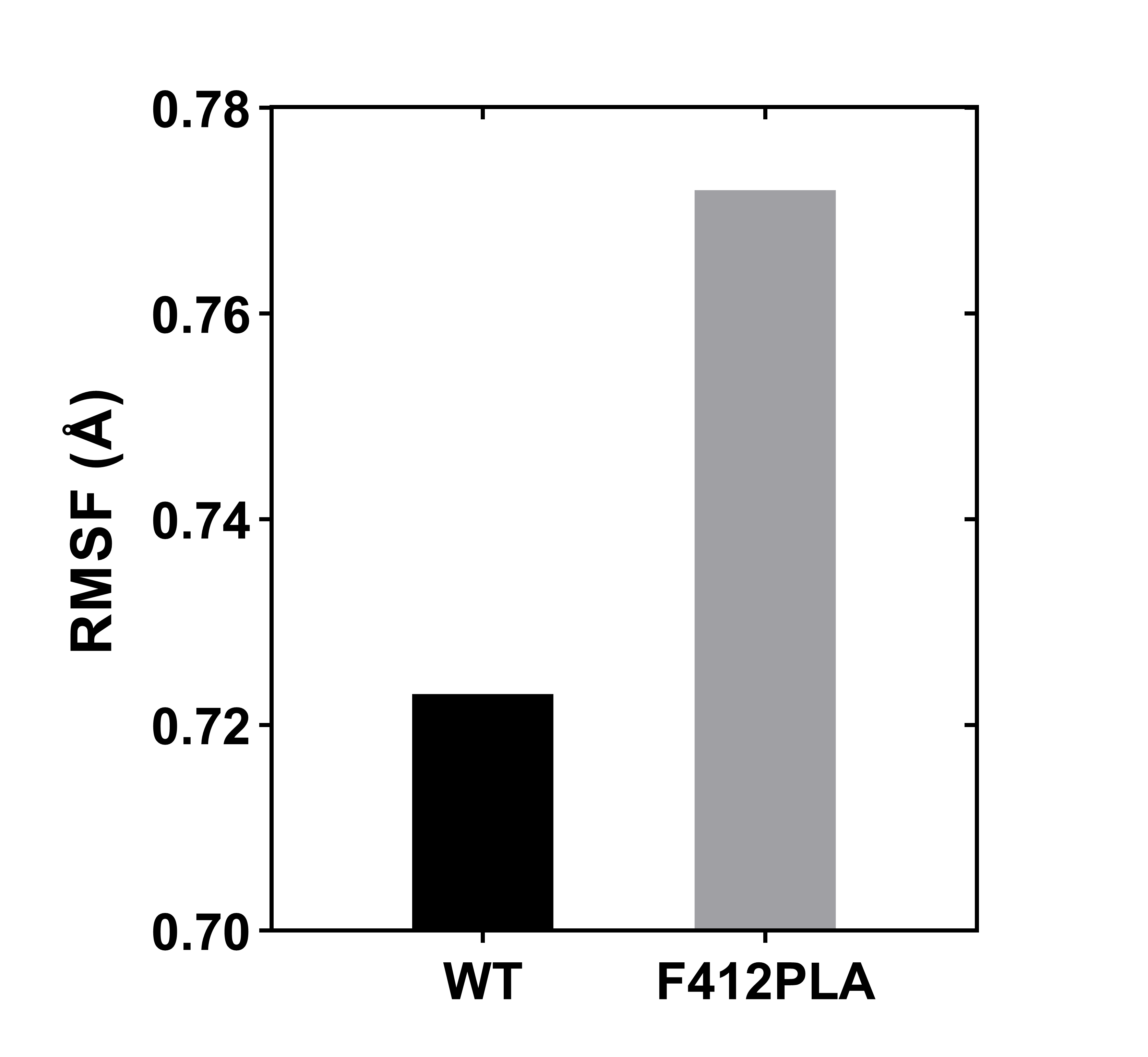


## Fig. S15 RMSF values of 412 site calculated for the backbone atoms of wild type and F412PLA mutant.

# Supplementary texts

## PedH mutants incorporated with ncAAs having *meta*-substituents

The mutants with the incorporation of Phe derivatives 3-I-Phe (**1**), 3-Br-Phe (**2**), and 3-Me-Phe (**3**) having *meta*-substituents showed the most significant reduction in activity. In particular, the incorporation of **1** led to almost complete loss of activity towards all substrates. To investigate the exact mechanism by which the *meta*-substituents at F412 site affect the enzyme catalysis, the substrate ethanol was docked into the wild-type PedH. The hydroxyl group of the substrate ethanol is close to the coenzyme PQQ's C5, catalytic residue D323, and the Pr^3+^ ion, consistent with the initial conformation of the lanthanide-dependent alcohol dehydrogenase reaction (Fig. S11A) (Prejano et al. 2020). The Chimera software was used to predict the residue conformation of ncAAs after incorporated at the site of 412, with the structures and force field files of ncAAs from the SwissSidechain database (Pettersen et al. 2004; Gfeller et al. 2013). After the Phe at 412 site was mutated to 3-Me-Phe **3**, the orientation of the residue side chain changed slightly, with a distance of only 2.4 Å between the methyl carbon atom of **3** and the α-carbon atom of substrate, which may result in substrate deviation from the reaction conformation during actual enzyme catalysis (Fig. S11B). Since force field files for **1** and **2** were unavailable in the SwissSidechain database, 3-fluoro-L-Phe and 3-chloro-L-Phe showing similar properties were selected for study. The fluorine atom of 3-fluoro-L-Phe and the chlorine atom of 3-chloro-L-Phe were 2.5 Å and 2.2 Å away from the substrate's α-carbon atom, respectively (Fig. S11C&D). It is conceivable that the distances between bromine or iodine atoms with larger radii and the substrate's α-carbon atom would be shorter, further decreasing the probability of the substrate being in the reaction conformation during enzyme catalysis. The activity of the three PedH mutants with *meta*-substituted groups of **3**, **2**, **1** decreased in order as the steric hindrance of the substituent increased, indicating that the steric hindrance of the *meta*-substituted group may directly affect the substrate binding.

## PedH mutants incorporated with ncAAs having *para*-substituents

Compared to the ncAAs with substituted groups at *meta*-position, the 412 site tolerates ncAAs with larger substituted groups at *para*-position, as evidenced by higher activities possessed by variants with *O*-benzyl-L-tyrosine (**7**), *O*-tert-butyl-L-tyrosine (**8**) and *O*-methyl-L-tyrosine (**9**) incorporated than those incorporated with 3-I-Phe (**1**), 3-Br-Phe (**2**), and 3-Me-Phe (**3**). Additionally, it was interesting to find that the enzyme activity seemed to decrease along with the size of substituted groups at the *para*-position as the *O*-methyl-L-tyrosine (**9**) with smallest size among ncAAs **7-9** showed the lowest activity. Moreover, the incorporation of *O*-benzyl-L-tyrosine (**7**) and *O*-tert-Butyl-L-tyrosine (**8**) changed the enantioselectivity of PedH which showed higher activity towards (*S*)-2-butanol than (*R*)-2-butanol, while the wild type exhibited similar activity against the two substrates. Kinetic parameters of wild type and variant F412OBT exhibited that the variant decreased the *K*_m_ values against (*S*)-2-butanol and (*R*)-2-butanol compared to the wild type (Table S1). The variant showed a *K*_m_ of 0.22 mM towards (*R*)-2-butanol lower than 0.39 mM of (*S*)-2-butanol, and a *k*_cat_ of 0.31 s^-1^ towards (*S*)-2-butanol higher than 0.17 s^-1^ of (*R*)-2-butanol (Table S1). These indicated that F412OBT variant exhibited an increased binding affinity towards (*R*)-2-butanol compared to (*S*)-2-butanol, and an improved turnover number against (*S*)-2-butanol relative with (*R*)-2-butanol.

MD simulations showed that (*S*)-2-butanol and (*R*)-2-butanol maintained different interactions with the PedH variant F412OBT. In the structure of F412OBT-(*R*)-2-butanol complex, (*R*)-2-butanol was found to form a hydrogen bond with Trp285, that was neither observed in the wild type nor F412OBT-(*S*)-2-butanol complex (Fig. S12A-D&S13). Additionally, the Trp561, a substrate binding residue in active center, in the F412OBT-(*R*)-2-butanol complex lost hydrogen bonds with Ala557 and Val558, that were observed in the wild type and F412OBT-(*S*)-2-butanol complex (Fig. S13). Moreover, due to the incorporation of *O*-tert-Butyl-L-tyrosine (**8**), oxygen of (*S*)-2-butanol was found to exhibit a shorter distance with C5 of PQQ compared to the (*R*)-2-butanol, while the two substrates had a similar distance in the wild type (Fig. S12E&F). Based on the reaction catalysis mechanism, the first step of the reaction is that the oxygen of the butanol attacks the C5 of PQQ and simultaneously donates its proton to the coordination shell residue, Asp323. The shorter distance might hence contribute to the enhanced activity, which might explain the higher activity of the variant towards (*S*)-2-butanol than (*R*)-2-butanol.

## PedH mutants incorporated with hydroxy acid

In order to better understand the effect of phenyllactic acid (**25**) on enzyme catalysis, kinetic parameters of F412PLA variant with phenyllactic acid incorporated against the substrate methanol were determined (Table S1). Compared with the wild type, the *K*_m_ value of F412PLA decreased from 1.10 mM to 0.70 mM, and the *k*_cat_ value increased from 0.15 s^−1^ to 0.17 s^−1^, which led to a 1.8-fold improved *k*_cat_/*K*_m_ for the variant compared to the wild type. The kinetic parameters showed that the incorporation of phenyllactic acid increased the affinity and turnover number of methanol, indicating that the subtle transition from peptide bond to ester bond in the main chain indeed improved the catalytic performance of the mutant towards methanol. The interaction between site F412 and surrounding residues was explored to investigate the impact of this subtle transition. MD simulations revealed that a hydrogen bond existed between the sulfur atom of M439 and the nitrogen atom on the main chain of F412, which would inevitably be disrupted by introducing phenyllactic acid (Fig. S14A&B). A hydrogen bond was also formed between the main chain oxygen atom of F412 and R350 in the wild type, which was lost in the mutant F412PLA. Phe412 maintained a single conformation in the MD simulations of wild type, whereas two conformations of PLA412 were observed in the mutant due to the breakage of these interactions (Fig. S14A&B). Distance between 412 site and 561 site was stably maintaining at around 4 Å for wild type in the MD simulations, while that of the mutant dramatically fluctuated, reflecting the higher dynamics of the PLA412 in the mutant than wild type, consistent with the calculation of the RMSF value (Fig. S14C&S15). Therefore, we hypothesized that the introduction of a phenyllactic acid disrupted the hydrogen bond between main chain of F412 and side chain of M439, which perturbed the hydrogen bond interaction network and dynamics of active sites, thereby affecting enzyme activity.

# References

Gfeller D, Michielin O, Zoete V (2013) SwissSidechain: a molecular and structural database of non-natural sidechains. Nucleic Acids Res 41 (D1):D327-D332. <https://doi.org/10.1093/nar/gks991>

Pettersen EF, Goddard TD, Huang CC, Couch GS, Greenblatt DM, Meng EC, Ferrin TE (2004) UCSF Chimera--a visualization system for exploratory research and analysis. J Comput Chem 25 (13):1605-1612. <https://doi.org/10.1002/jcc.20084>

Prejano M, Russo N, Marino T (2020) How Lanthanide Ions Affect the Addition-Elimination Step of Methanol Dehydrogenases. Chem-Eur J 26 (49):11334-11339. <https://doi.org/10.1002/chem.202001855>
